# Cross-Source Prediction of a Visual Load Index from Vehicle Kinematic Features: A Driver-Independent Validation Study

**DOI:** 10.3390/s26175521

**Published:** 2026-08-31

**Authors:** Tursun Mamat, Siyi Cheng, Chunguang He, Jiake Wuyuncaicike

**Affiliations:** 1 School of Transportation and Logistics Engineering, Xinjiang Agricultural University, Urumqi 830052, China; 2 Intelligent Transportation Engineering Research Center, Xinjiang Agricultural University, Urumqi 830052, China; 3 Xinjiang Key Laboratory of Transportation and Logistics Engineering, Xinjiang Agricultural University, Urumqi 830052, China

**Keywords:** visual load index, vehicle kinematics, cross-source prediction, naturalistic driving, driver monitoring systems

## Abstract

**Highlights:**

**What are the main findings?**

- Across held-out drivers, vehicle kinematics retained measurable information about the eye-movement-derived visual load index (VLI) proxy beyond the road-type baseline (ΔAUC = 0.042; 95% CI: 0.006–0.078).
- The driving-style prior increased AUC from 0.589 to 0.633, but its driver-level incremental effect remained exploratory because the confidence interval included zero.

**What are the implications of the main findings?**

- By integrating within-fold proxy-label construction, source isolation, driver-grouped evaluation, and road-context controls, this study establishes a confounder-aware framework for identifying transferable information in proxy-label-based multisource sensing research.
- These findings position vehicle kinematics as a complementary sensing channel, rather than a replacement for eye tracking, and support its prospective evaluation in multisource driver-monitoring systems.

**Abstract:**

Continuous eye tracking is difficult to deploy in production vehicles, while eye-movement-derived proxy labels are susceptible to same-source circularity and road-type confounding. We propose a driver-independent, confounder-aware cross-source framework in which the eye-movement-derived visual load index (VLI) proxy is constructed within training folds, whereas held-out prediction uses only vehicle kinematics and an optional driving-style prior. Evaluation used naturalistic data from 33 drivers, comprising 24,437 windows along a 48.4 km route, under driver-grouped cross-validation with driver-level bootstrap confidence intervals and control and increment tests. The vehicle-only model achieved an AUC of 0.589 across held-out drivers, rising to 0.633 with the style prior. After controlling for road type, vehicle features improved AUC by 0.042 (95% CI: 0.006–0.078), indicating measurable information beyond the binary road-type proxy; the style increment remained exploratory because its confidence interval included zero. Future-state prediction converged to a persistence baseline at 20 s. The VLI was not externally validated against an independent subjective or physiological criterion; accordingly, the findings are limited to cross-source prediction of an eye-movement-derived proxy. Across five architectures, this cross-source pattern was directionally consistent, supporting prospective evaluation of vehicle kinematics as a complementary sensing channel in multisource driver-monitoring systems.

## 1. Introduction

Driver state monitoring is central to intelligent transportation and vehicle active safety: excessive visual–cognitive load strains a driver’s limited attentional resources, delays hazard perception and control responses, and is a major human factor in road traffic crashes. As sensing and computing capabilities in production vehicles grow, continuously monitoring driver load non-intrusively and integrating it into driver-monitoring systems (DMSs) has become a practical requirement. Eye movements provide a widely used nonintrusive source of load-related behavioral indicators, but their acquisition depends on dedicated eye trackers whose cost, calibration, and wearing requirements hinder large-scale deployment. Vehicle kinematic signals such as speed, acceleration, and heading change can instead be acquired continuously and cheaply by on-board sensors, yet lack a direct measurement correspondence with visual load. This contrast raises a question of both practical and methodological significance: can visual load labels derived from eye movements be predicted from non-ocular sources such as vehicle kinematics under driver-independent and confound-controlled conditions? This cross-source measurement validity question is the focus of the present study.

The link between eye-movement metrics and driver mental workload has a long research tradition. In a review, Marquart et al. reported that blinks, fixation duration, saccadic characteristics, and pupillary changes all vary with task demand [1]. Recarte and Nunes showed that verbal and spatial tasks alter fixation patterns in different ways and that mental workload substantially affects visual search, discrimination, and decision making [2,3]. Benedetto et al. and May et al. further employed blink duration and eye-movement indices as representations of mental workload [4,5]. Together, these studies provide the basis for constructing a visual load index (VLI) from window-level eye-movement statistical features (WESFs). They also suggest, however, that eye movements should be regarded as load-related evidence rather than cognitive load itself, and that their sensitivity depends on task structure and scenario conditions. In naturalistic driving, eye movements are additionally shaped by road geometry, traffic flow, speed control, and individual habits. Accordingly, this study defines the VLI as a proxy label constructed through combined entropy-weight and CRITIC weighting, and interprets model performance as the reconstruction of this constructed label space rather than as validation against external workload scales or physiological annotations.

This study focuses on two road categories, urban expressways and freeways, which differ markedly in alignment standard, ramp density, and traffic organization, with entrances, exits, and merging or diverging ramps densely distributed along urban expressways. Road geometry and traffic organization jointly shape both vehicle kinematics and driver load, and this mechanism is directly supported by existing evidence: Xu et al. characterized vehicle trajectory features at high-density interchange ramp terminals and showed that road geometry and merge–diverge configurations markedly alter the kinematic response of vehicles [6], while Mu et al. found, using heart rate variability, that insufficient ramp spacing elevates driver mental load at comparable terminals [7]. Thus, vehicle kinematics themselves carry road-context information that motivates predicting load from the vehicle side, and high-density ramp sections are scenarios in which driver load frequently peaks. Zhang et al. further corroborated the substantial influence of dense merging and diverging on visual–cognitive demand through on-road experiments in a high-density interchange merging area [8]. Consistent with this, Shahbakhti et al. combined electroencephalography (EEG) with eyelid and eye-movement features for drowsy-driving detection and confirmed that eye-movement-related information can effectively characterize driver state; because their method extracts blink signals directly from the same frontal EEG channel, it highlights the same-source coupling among such signals [9], providing a practical rationale for strictly separating eye-movement-derived VLI labels from vehicle-kinematics-based prediction to avoid same-source circularity.

Beyond eye movements, cognitive demand can also be reflected in other physiological or behavioral signals. Mehler et al., Borghini et al., and Brookhuis et al. examined, respectively, the sensitivity of physiological indices to cognitive demand, the assessment of load and fatigue in pilots and drivers, and the monitoring of mental workload in simulators [10,11,12], suggesting that although a single ocular source is effective, it may carry same-source bias. Eye-movement-based load modeling also faces a transfer challenge from simulators to real roads, and previous work has shown that high accuracy in one environment does not necessarily generalize to real-road conditions [13,14,15]. Accordingly, this study uses naturalistic driving data together with driver-grouped cross-validation to bring the generalization assessment closer to practical application. The use of machine learning for cognitive load recognition has likewise been explored extensively [16,17,18,19]. Taken together, these studies indicate that multisource signals help improve the completeness of interpretation, whereas scenario and individual differences alter the meaning of load indicators.

Research on driving style provides a basis for introducing a driver-level prior. Measurement studies based on multidimensional driving-style scales, together with behavioral studies relating crash risk to individual differences, indicate that drivers are not interchangeable random samples and that their control habits and maneuvering patterns may remain stable over the long term [20,21,22]. Sensor and machine-learning studies further show that driving style can be identified from smartphones, vehicle trajectories, or controller area network (CAN) bus data [23,24,25,26]. This supports constructing a driver-level style vector from statistics of speed, acceleration, lateral control, and heading change, but it does not guarantee that a style prior will necessarily improve the reconstruction of the VLI from WESFs. Naturalistic driving data, in turn, provide a realistic basis for driver-independent validation [27,28,29,30,31,32,33]. Window-level samples are not mutually independent units of generalization, and driver-level held-out evaluation is what determines the credibility of naturalistic driving research.

At the model level, extreme gradient boosting (XGBoost, v2.0.3) [34], attention-based Transformer [35], gated recurrent unit (GRU) [36], and random forest (RF) [37] are used only as tools for consistency checking and probability-stream generation, with post hoc attribution provided through a unified interpretation framework [38], rather than being stacked as an architectural innovation. Although recent sensor and multimodal-learning research often models multi-source signals through parallel branches, attention mechanisms, and gated fusion, a multi-sensor combination is not necessarily superior to a single source, and when the label itself is constructed from eye-movement features, using those same features as the predictive input creates same-source circularity. For this reason, the primary predictive model in this study does not use any ocular input, joint eye–vehicle input, or any fusion setup containing an ocular branch, and comparisons are made only among the Vehicle-only, Vehicle+Style, and road-control models; the core contribution remains the design itself, namely proxy-label construction, input-source delimitation, and road-confounding diagnosis.

In parallel, positioning this study against recent workload-estimation research clarifies the gap it addresses. Recent studies have advanced driver workload and state estimation from ocular, physiological, and driving-behavior signals [39,40,41,42], including Bayesian workload estimation from on-road driving-performance data [39], dual-stage and stacking-based learning approaches [40,42], and interpretable multimodal classification frameworks [41]. Three methodological gaps nevertheless persist across this line of work. First, controlled studies obtain workload labels through task manipulation or dedicated probe tasks in simulators or instrumented drives [39,41], but such labels are unavailable in naturalistic driving, where labels must instead be constructed from sensor measurements—typically ocular or physiological—so that a same-source circularity risk arises whenever the label-defining sensors also contribute predictive features, a risk that is rarely diagnosed explicitly. Second, evaluation is often not driver-independent: subject-mixed cross-validation allows individual-specific information to enter both training and test partitions, a limitation acknowledged in recent work [41]. Third, the road and route context that jointly shapes vehicle kinematics and driver load is seldom quantified or entered as an explicit control [27,28,29,30,31,32,33], leaving open the possibility that apparent predictive power reflects a road proxy. To our knowledge, no prior study combines within-fold proxy-label construction, driver-grouped cross-validation, and explicit road-confounding diagnosis in a single framework; closing this joint gap is the aim of the present study.

The main contribution of this study is a confounder-aware framework for assessing whether vehicle kinematics retain cross-source information about an eye-movement-derived VLI proxy across held-out drivers. The framework comprises the following four elements. (1) A within-fold VLI label construction procedure is proposed, in which eye-movement statistical features are strictly confined to the label-construction stage, blocking same-source circularity at the outset. (2) A driver-grouped cross-validation scheme with driver-level bootstrap confidence-interval estimation is established, preventing overlapping windows from being mistaken for independent samples. (3) A fair comparison system is built between the vehicle and style predictive models and the road-control model, complemented by within-road stratification and within-road relabeling diagnostics to isolate the road-type proxy effect. (4) Positive and negative controls are introduced to distinguish apparent accuracy from measurement validity. The framework therefore distinguishes three common validity threats in proxy-label studies: same-source circularity, driver leakage, and contextual confounding. The prediction target remains an eye-movement-derived VLI proxy rather than an independently validated visual-load construct.

## 2. Materials and Methods

### 2.1. Data Acquisition and Preprocessing

#### 2.1.1. Experimental Equipment and Sensor Configuration

**Figure 1:**
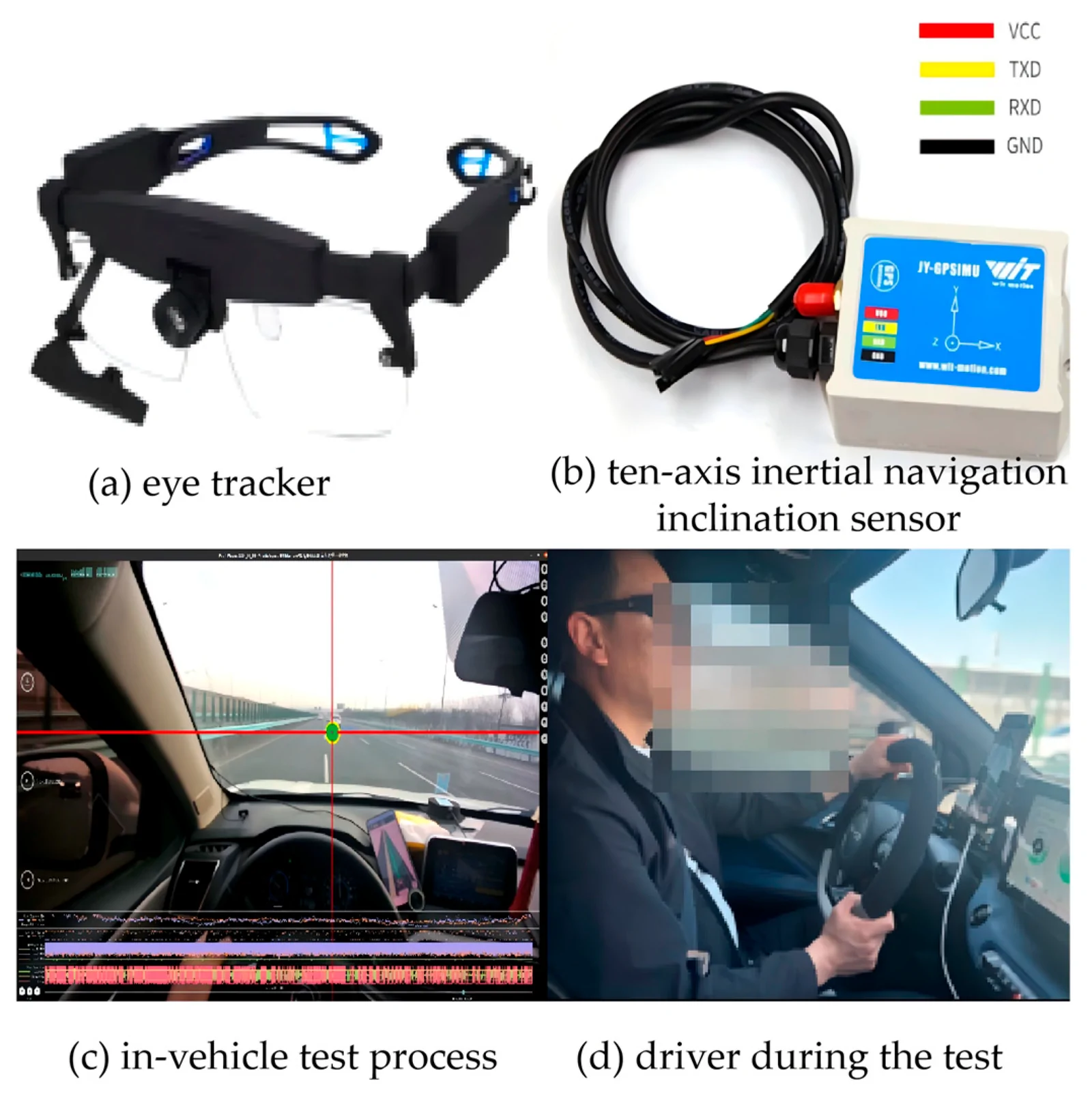
Experimental equipment and setup in the naturalistic driving test: (**a**) eye tracker; (**b**) ten-axis inertial navigation inclination sensor; (**c**) in-vehicle test process; (**d**) driver during the test.

Driver eye-movement data were collected with Eyeso Glasses eye trackers (Braincraft Technology (Beijing) Co., Ltd., Beijing, China), which record visual-behavior indicators such as fixations, saccades, pupil size, and gaze-point location. Vehicle kinematic data were synchronously acquired by a high-precision inertial navigation system (WTGAHRS3-TTL/232, WitMotion Shenzhen Co., Ltd., Shenzhen, China), covering vehicle position, heading angle, speed, acceleration, and attitude change, and were used to reconstruct the driving trajectory as well as the longitudinal and lateral control states. The test vehicle was a BYD Seagull (BYD Auto Co., Ltd., Shenzhen, China), on which one dashcam (70mai Dash Cam A510, 70mai Co., Ltd., Shanghai, China) was mounted behind each of the front and rear windshields to record the traffic scenes ahead of and behind the vehicle, providing a video basis for timestamp calibration, review of anomalous segments, and annotation of road events. All features used in the modeling stage were anonymized derived quantities, including eye-movement statistics, vehicle-kinematic statistics, and road-scene encodings. The experimental equipment and the on-board sensor layout are shown in Figure 1, and the specifications of each sensor together with the signal-processing parameters are listed in Table 1.

As shown in Table 1, all sensor specifications meet the modeling requirements, with fixation and saccade segmentation performed by the I-VT algorithm at a 30°/s velocity threshold [43]. The time synchronization, quality control, and windowing procedures for each source are detailed in Section 2.2.

#### 2.1.2. Participants

A total of 33 drivers were recruited for this experiment, all of whom completed data acquisition, yielding one driver-level modeling dataset for each of the 33 participants. The participants were aged 24–50 years (mean 35.0 years) and had 3–20 years of driving experience (mean 8.0 years). All participants held a valid driver’s license and had normal or corrected-to-normal visual acuity meeting the requirements for safe driving. Before the experiment, participants were required to ensure adequate sleep and to be in good physical condition, and they were allowed to take part only after being confirmed capable of completing the naturalistic driving task safely.

Prior to the start of the experiment, the purpose, procedure, potential risks, and data-use arrangements were explained to the participants; all participants took part voluntarily and signed an informed-consent form. No secondary task was imposed at any time, and participants performed naturalistic driving according to their everyday driving habits. The collected data were used solely for scientific analysis and were anonymized during data curation to protect participant privacy.

#### 2.1.3. Experimental Scenario

**Figure 2:**
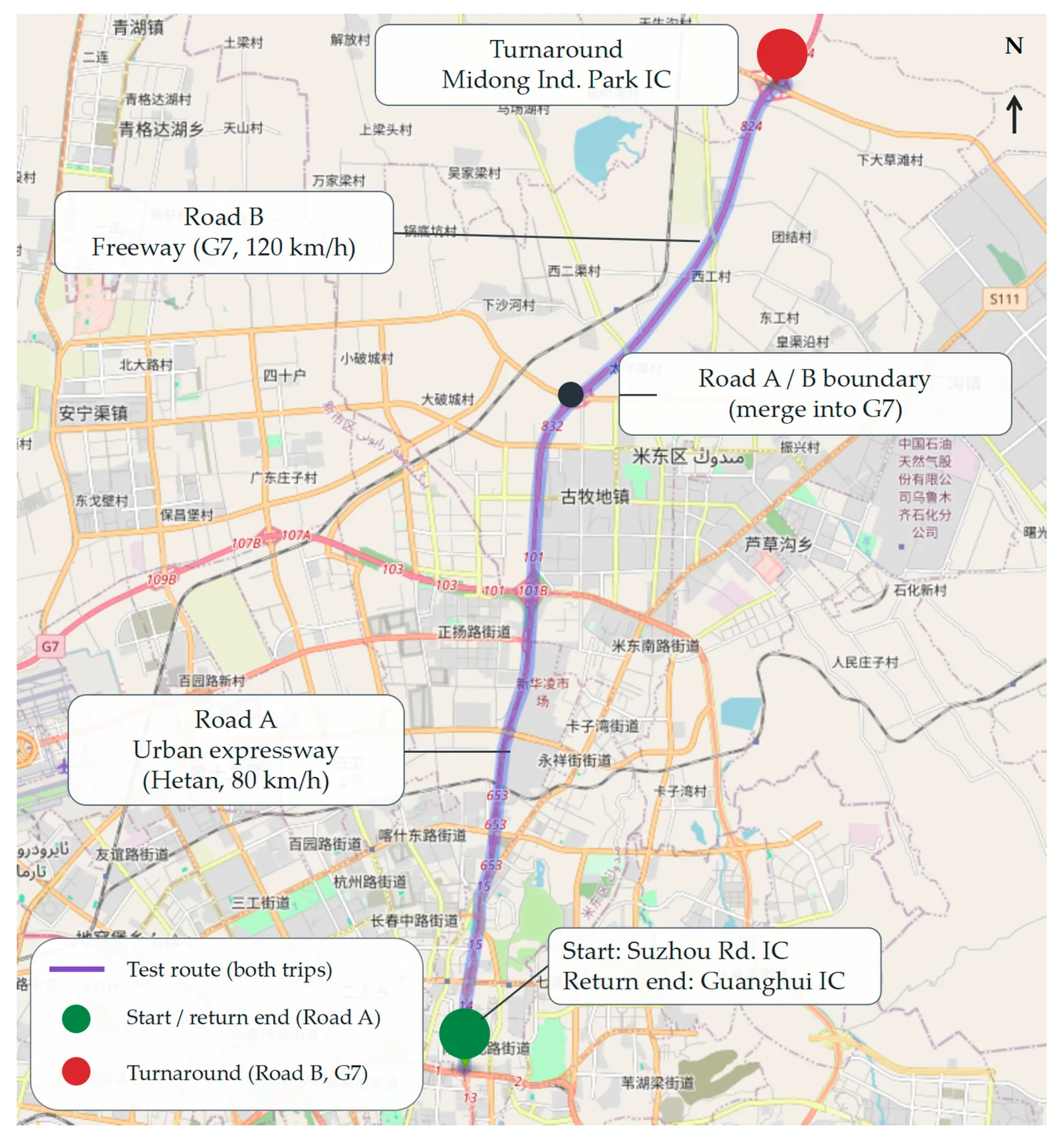
Naturalistic driving test route in Urumqi. The black dot marks the boundary between Road A and Road B (merge into the G7). The background place-name labels are rendered by the OpenStreetMap base map and are not required for interpreting the test route. Base map © OpenStreetMap contributors.

This study selected the section from the Hetan Expressway to the Midong Industrial Park interchange of the Beijing–Xinjiang Freeway (G7) in Urumqi as the naturalistic driving test route: starting at the Suzhou Road interchange entrance of the Hetan Expressway, proceeding northward along the mainline, merging into the G7 through a grade-separated interchange, turning around at the Midong Industrial Park interchange bridge, and returning along the same route to the Guanghui interchange, for a round-trip distance of 48.4 km. Multiple urban-expressway entrances and exits, grade-separated interchanges, and merging or diverging ramps are distributed along this section, so that the driver must continuously allocate visual attention and perform speed control, lane keeping, and trajectory planning according to sight distance, lane layout, and real-time traffic conditions. The diverse driving situations covered by this section elicit variations in driver visual–cognitive load, providing naturalistic variation within the sampled route. The naturalistic driving test route is shown in Figure 2.

The two road categories are defined by functional class and design standard. Road A is the Hetan Expressway, an urban expressway (per the Specification for Design of Urban Expressway, CJJ 129 [44]), operating under continuous flow at an 80 km/h limit with dense entrances, exits, and merging or diverging ramps. Road B is the G7 section of the Beijing–Xinjiang Freeway, a freeway (per the Technical Standard of Highway Engineering, JTG B01 [45]), fully access-controlled and grade-separated at a 120 km/h limit, with sparse entrances and a high alignment standard. The two are delimited by the physical boundary where the Hetan Expressway mainline merges into the G7 mainline through the grade-separated interchange: the mainline and connecting ramps south of this boundary are assigned to Road A and those to the north to Road B, with the same boundary applied to both trips. Their systematic differences in alignment standard, ramp density, and traffic organization provide a realistic basis for controlling road type as a confounding variable in Section 3.5.

#### 2.1.4. Data Acquisition Procedure

To reduce the effects of traffic congestion, extreme weather, and abnormal illumination on the eye-movement and vehicle-kinematic data, data acquisition was carried out under controlled conditions. The experiment was conducted during weekday daytime hours from 9:00 to 17:00, outside the principal morning and evening commuting peaks, when the weather was clear and visibility was good. Before each session, the experimental instructions and precautions were explained to the driver, who then put on the eye tracker and completed multi-point calibration; acquisition began only after the calibration error was confirmed to be within 1°. During formal acquisition, the in-vehicle infotainment system was switched off to eliminate audiovisual interference, and the driver drove according to their everyday habits, performing maneuvers such as lane changes and overtaking independently in response to real-time traffic conditions. An in-vehicle assistant recorded the start and end times of key road sections in real time and annotated events such as congestion, temporary stops, traffic accidents, construction detours, interference from anomalous vehicles, strong illumination, and brief eye-tracker inaccuracies, providing the basis for subsequent data cleaning.

To address the inconsistency in sampling frequency and start time across the multisource sensors, the acquired data were cleaned and fused in sequence: redundant data recorded outside the formal test route—during experiment preparation and post-test vehicle repositioning—together with occasional interference segments were removed by comparing the dashcam video with manually marked points; the visual sequences and the vehicle trajectory were synchronously registered on GPS time and resampled by cubic-spline interpolation; brief eye-movement gaps caused by blinks were filled by linear interpolation, whereas segments with continuous loss exceeding 500 ms due to prolonged eye closure were removed directly; and segments affected by strong illumination, abrupt shadow changes, window or preceding-vehicle reflections, and brief eye-tracker inaccuracies were reviewed one by one and removed, drawing on the video, on-site records, and the tracker’s validity flags. For the retained valid pupil data, individual baseline correction and standardization reduced individual differences, and moving-median filtering with outlier screening suppressed noise from transient illumination fluctuations; the speed and acceleration sequences were smoothed by moving-average filtering to remove high-frequency noise.

Based on the event log annotated in real time by the in-vehicle research assistant, four congestion events and two temporary-stop events were identified. These events lasted approximately 38 min in total, accounting for 1.8% of the raw data, and were excluded during data cleaning together with the other interference segments described above.

When the in-vehicle assistant identified substantial data loss during a session—prolonged eye-tracker signal degradation or external interference exceeding the pre-specified quality criteria—the affected driver completed a supplementary round-trip session under the identical protocol and within the same April 2025 acquisition window. Valid segments of the original sessions were retained together with the supplementary sessions; in total, the 33 drivers contributed 41 round-trip sessions (25 drivers completed one session and eight completed two). Supplementary sessions were triggered solely by these data-quality criteria, which were defined before any modeling analysis.

As summarized in Table 2, severely constrained windows accounted for only 1.3% of the retained sample, and the between-session coefficient of variation in one-way travel time was 7.3%. On both roads, mean speeds were lower for the full sample than for the basic segments, consistent with deceleration in ramp-influenced zones rather than congestion. Collectively, these results indicate that the retained sample predominantly captured non-congested, near-free-flow operating conditions.

#### 2.1.5. Analysis Samples and Driver-Independent Validation

The analysis samples were derived from the naturalistic driving data of 33 drivers. After time synchronization, outlier removal, and resampling to a unified 1 Hz, 122,411 valid time-series records were retained across all 41 sessions, corresponding to about 34.0 h of effective driving (mean 61.8 min per driver, range 47.8–81.3 min). The continuous driving process was then segmented with a 10 s sliding window at a 5 s step (50% overlap), and eye-movement statistical features, vehicle-kinematic statistical features, and the road-type encoding were computed within each window, yielding 24,437 window-level analysis samples. The window-level road-type encoding was assigned after map-matching the within-window GPS trajectory points to the two road categories of Section 2.1.3; a window crossing the Road A/B boundary or lying on an interchange connecting ramp was assigned to the road containing its midpoint.

For the spatial sensitivity analyses in Section 3.5, each window was assigned, in addition to its road-type code, to one of four mutually exclusive categories. Ramp-influenced windows were operationally defined as those whose midpoint fell within 300 m upstream or downstream of a merge or diverge gore nose. When adjacent 300 m influence zones overlapped, they were partitioned at the midpoint between the two gore noses, such that each location was assigned to one of the adjacent ramp-influenced zones. The test route contained no formal weaving segments in which a continuous auxiliary lane connected an entrance ramp to a downstream exit ramp. Where a merge gore nose was closely followed by a diverge gore nose, the adjacent influence zones jointly covered the intervening mainline. These intervening sections were therefore included in the ramp-influenced category rather than treated as a separate weaving category. Connecting-ramp windows were defined as those whose midpoint lay on an interchange connecting ramp, whereas boundary-crossing windows contained GPS trajectory points from both road categories. All remaining windows were classified as basic-segment windows on Road A or Road B. The 300 m buffer was adopted as a working definition and was comparable in magnitude to previously reported ramp influence areas. In a field study of comparable urban interchanges, approximately 150 m of the mainline upstream of the merge nose was defined as an influence segment [8], whereas the Highway Capacity Manual specifies an influence-area length of 1500 ft, approximately 450 m, at ramp junctions [46]. The sensitivity-analysis results remained unchanged when the buffer distance was varied from 150 to 450 m. This classification was used only as an additional annotation layer for the appendix sensitivity analyses; the road-type encoding in the primary analysis continued to follow the midpoint-assignment rule described above. Of the retained windows, 87 (0.4%) crossed the Road A/B boundary and 350 (1.4%) lay on connecting ramps.

In addition, each window was labeled as either outbound or return according to its temporal position within the session relative to the turnaround at the Midong Industrial Park interchange, and the resulting labels were cross-validated against the GPS heading. Windows recorded during the turnaround maneuver corresponded to connecting ramps and were therefore subsumed under the spatial classification described above. These auxiliary spatial and directional labels were used only for the sensitivity analyses reported in Section 3.5.

For the maneuver-level analysis, the retained windows were assigned to one of three mutually exclusive categories: windows involving merging or diverging maneuvers, windows involving lane-changing or overtaking maneuvers, and regular-driving windows. Windows within the ramp-influenced zones defined above were classified as involving merging or diverging maneuvers. Among the remaining non-ramp windows, candidate windows involving lane-changing or overtaking maneuvers were identified using the joint kinematic trigger defined in Equation (1):

(1) $$\begin{array}{l} m_{a}(\omega )=\underset{t\in \omega }{\max}\left|a_{y}(t)\right|,\\ m_{\omega }(\omega )=\underset{t\in \omega }{\max}\left|\omega_{z}(t)\right|,\\ C_{LCOT}^{cand}(\omega )\left\{\begin{array}{l} I\left[m_{a}(\omega )>P_{90}^{train}m_{a}\wedge m_{\omega }(\omega )>P_{90}^{train}m_{\omega }\right],\omega \notin R\\ 0,\omega \in R \end{array}\right. \end{array}$$

where $m_{a}(\omega )$ and $m_{\omega }(\omega )$ denote the window-level maxima of $\left|a_{y}\right|$ and $\left|\omega_{z}\right|$, respectively, and $P_{90}^{train}m_{\omega }(\cdot )$ denotes the 90th percentile of the corresponding window-level statistic, estimated exclusively from training-fold windows outside the ramp-influenced zones.

In naturalistic driving data, adjacent windows from the same driver are highly continuous in eye-movement baseline, road exposure, and vehicle-control habits, so windows cannot be treated as mutually independent units of generalization: a random window split would let a model overestimate its out-of-fold performance by memorizing a specific driver’s baseline, speed preference, control habits, or road-exposure pattern. This study therefore adopts five-fold driver-grouped cross-validation with the driver as the smallest partition unit. All windows of a held-out driver are used only for out-of-fold testing; the normalization parameters, the entropy and CRITIC weights, the quantile thresholds, the driving-style cluster centers, and the model architecture and hyperparameters are all estimated solely on the training-fold drivers’ data (the number of style clusters, *K* = 3, was fixed before the modeling analysis on the basis of the whole-sample descriptive diagnostics in Section 3.2 and was not re-selected within folds), then frozen and applied to window-level prediction and performance evaluation on the held-out drivers. This procedure controls the risk of driver-level information leakage at its root and brings the performance estimate closer to the true predictive capability expected when the model is deployed to new drivers. Because eight drivers contributed two sessions (Section 2.1.4), all sessions of a given driver were assigned to the same fold (fold composition in Table A7), and driver-level style features were computed by pooling all sessions of each driver; supplementary sessions therefore introduced no cross-fold leakage.

Road type is both an important descriptive variable of the naturalistic driving scenario and a potential confounder: the two road categories differ systematically in speed level, lane conditions, traffic flow, lateral interference, and visual-search demand, and a model might misinterpret differences in road operation as differences in the VLI label. Therefore, in interpreting the results, this study does not equate the road-type effect with a change in visual load itself but examines it through road-control input settings (M0, M3, and M4, defined in Section 2.5) and within-road relabeling diagnostics, as detailed in Section 3.5.

**Figure 3:**
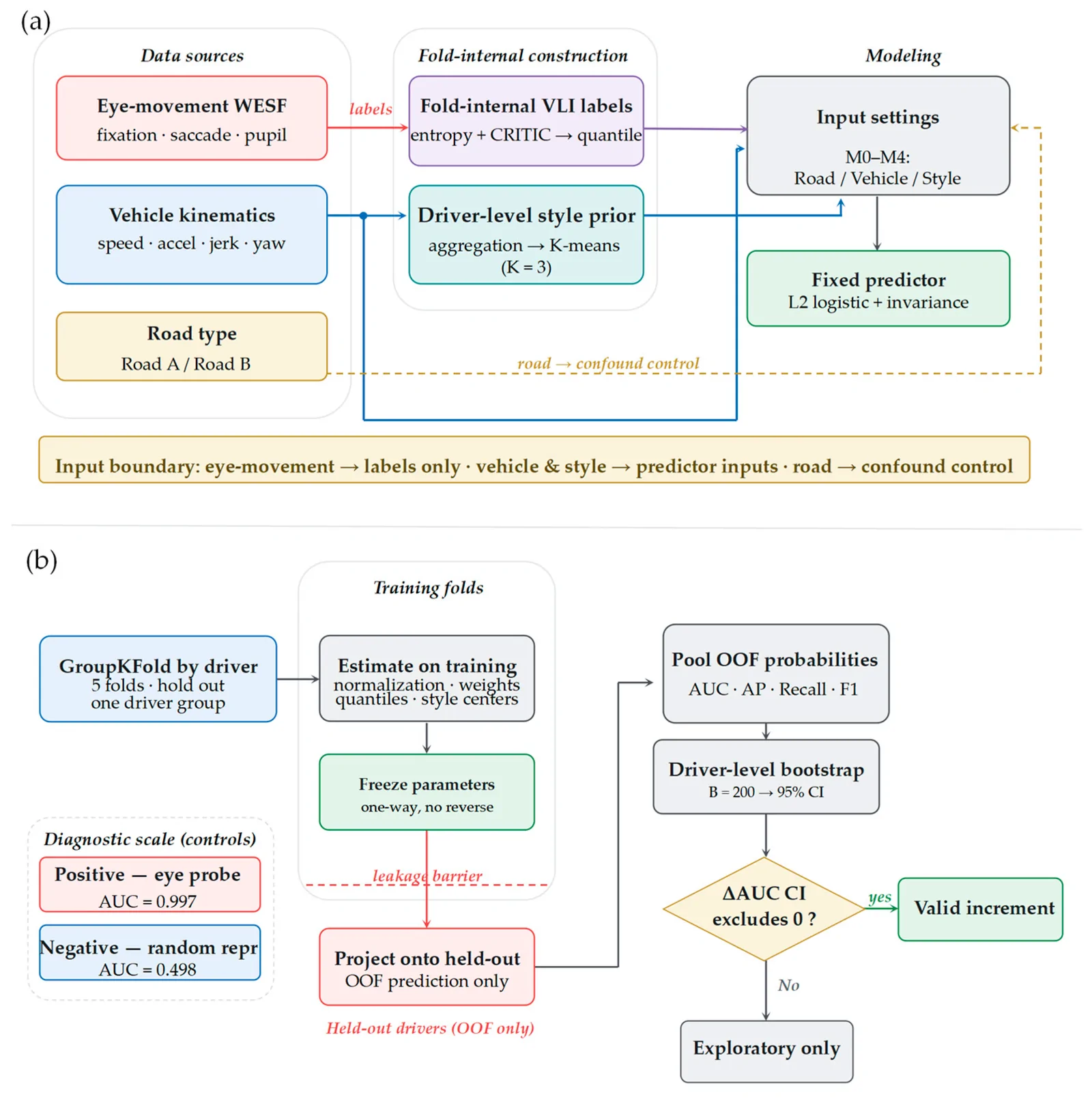
Role-separated cross-source pipeline and driver-grouped evaluation: (**a**) overall framework; (**b**) evaluation and leakage control. Colored arrows denote data pathways: red, label construction; blue, predictor inputs; the yellow dashed arrow, road-confounding control; and the red dashed line, the information-leakage barrier between training and held-out folds.

The overall research workflow of this study, covering label construction, cross-modal transfer, driver-grouped evaluation, and information-leakage control, is summarized in Figure 3.

### 2.2. Data Synchronization, Quality Control, and Windowing

Building on the multisource cleaning and time alignment described in Section 2.1, this study established a unified 1 Hz time axis on a per-driver basis, so that the eye-movement, vehicle-kinematic, and road-scene-derived features shared a consistent temporal reference at the sample level. After alignment, validity-mask construction, robust pupil baseline correction, sliding-window segmentation, and window-level vehicle-kinematic feature extraction were performed in sequence, as detailed below.

Under conditions such as blinks, strong illumination, occlusion, and brief inaccuracies, the eye-movement data contain invalid records; if these entered the window statistics directly, the pupil and fixation features would be corrupted by outliers. To address this, a valid-data mask was constructed that retained only the sampling points at which eye tracking, video review, and vehicle data were all valid, and the set of valid sampling times for each driver was determined accordingly.

Pupil indices are influenced by both the individual baseline and ambient illumination. To reduce differences in pupil-baseline level across drivers, robust baseline correction of pupil diameter was performed within each driver’s valid trip, as given in Equation (2); the scale term uses the median absolute deviation (MAD) rather than the standard deviation, to lessen the influence of blink residuals and transient illumination fluctuations on the scale estimate.

(2) $$p_{d}^{\mathrm{rob}}(t)=\frac{p_{d}(t)-{\mathrm{median}}_{\tau \in T_{d}^{+}}p_{d}(\tau )}{{\mathrm{MAD}}_{\tau \in T_{d}^{+}}p_{d}(\tau )+\varepsilon_{p}},\;t\in T_{d}^{+}$$

where $p_{d}^{\mathrm{rob}}(t)$ is the robust baseline-corrected pupil diameter of driver *d* at time *t*; $p_{d}(t)$ is the raw pupil diameter; $T_{d}^{+}$ is the set of valid sampling times of driver *d*; and $\varepsilon_{p}$ is a small constant for numerical stability. This correction serves only to reduce pupil-baseline differences across drivers and involves no load-label information.

To balance the temporal resolution of short-term states against the stability of the window statistics, overlapping time-series samples were constructed using a sliding window with a 10 s length and a 5 s step. The *k*-th window of driver *d* and its valid sampling subset are defined in Equation (3).

(3) $$\begin{array}{l} W_{d,k}=\{t\in T_{d}|t_{d,0}+(k-1)\Delta_{\mathrm{step}}\leq t<t_{d,0}+(k-1)\Delta_{\mathrm{step}}+L\},\\ W_{d,k}^{+}=\{t\in W_{d,k}|M_{d}(t)=1\},\\ n_{d,k}=\left|W_{d,k}^{+}\right| \end{array}$$

where $W_{d,k}$ is the *k*-th window of driver *d*; $t_{d,0}$ is the start time of that driver’s valid trip; $M_{d}(t)$ is the validity mask, taking the value 1 when the eye-movement, video, and vehicle data are all valid at that instant and 0 otherwise; $W_{d,k}^{+}$ represents the valid samples retained within the window; $n_{d,k}$ denotes the number of valid samples; *L* is the window length, set to 10 s; and Δ_step_ is the window step, set to 5 s.

Longitudinal jerk was obtained as the first-order difference of longitudinal acceleration between adjacent instants on the 1 Hz time axis; it characterizes the instantaneous fluctuation of longitudinal control and the smoothness of acceleration and deceleration, and it serves as a candidate feature for the vehicle modality and the driving-style prior.

After window segmentation, vehicle-kinematic signals were converted into window-level statistical representations using a unified aggregation strategy. For each vehicle variable *u_d_*(*t*), five statistical descriptors, including the mean, standard deviation, maximum, minimum, and median values, were calculated within each window to construct the vehicle-based feature representation for subsequent modeling. The definitions and physical units of all extracted features are summarized in Table 3.

The unified 1 Hz time base was adopted for three reasons. In terms of multisource alignment, 1 Hz is the resolution at which the 60 Hz eye-movement stream, the inertial stream, and the video-derived annotations can be jointly aligned on a single GPS-referenced time axis. In terms of feature construction, all vehicle-side features are 10 s window-level statistics—the mean, standard deviation, extrema, and median—rather than event-level measures, so the analysis concerns the window-scale smoothness and variability of vehicle control; for this purpose, second-scale sampling of the underlying signals constitutes the operative resolution, and the exact amplitudes of sub-second transients are not the analytical target. Moreover, window-level aggregation of vehicle kinematics at approximately 1 Hz is established practice in naturalistic driving research [27,28,29,30,31,32,33]. It should nevertheless be noted that resampling to 1 Hz attenuates sub-second transients in jerk, angular velocity, braking, and lane changing; the window-level statistics of jerk and angular velocity should therefore be interpreted as low-frequency envelopes of the underlying dynamics, and the reported discriminative performance as a conservative estimate of the information carried by the vehicle modality.

The window-level features fall into three groups with strictly separated roles. The first group comprises the nine WESFs, which cover fixation, saccade, and pupil-diameter statistics and are listed with their weights in Table 4; these features are used exclusively for VLI label construction. The second group consists of the vehicle-kinematic statistics summarized in Table 3, which serve only as predictor inputs to the model. The third group is the road-type encoding, which is used solely for confounding control. By assigning each feature group a single explicit role, this usage boundary blocks the same-source pathway between label construction and model prediction at the feature level.

### 2.3. VLI Proxy-Label Construction via Entropy–CRITIC Weighting

To characterize a driver’s short-term visual state without relying on external annotations, this study constructed a visual load index proxy label from eye-movement statistical features. Rather than serving as a gold standard of true cognitive load, the VLI is an operational label derived through feature alignment, objective weighting, and discretization. The aim of this study is therefore to examine whether this derived label can be effectively reconstructed from non-ocular modalities such as vehicle kinematics. To prevent data leakage, all parameters involved in VLI construction were estimated exclusively within the training folds, and the held-out drivers were excluded from the fitting process.

The nine WESFs were selected because prior studies have reported load-sensitive behavior in their respective pupillary, fixation, and saccadic feature families [1,2,3,4,5]. Combined entropy–CRITIC weighting was used to derive aggregation weights from the training-fold distributions and reduce reliance on subjective weight assignment. The resulting VLI is therefore a literature-informed composite of ocular indicators rather than an arbitrarily specified index. Its empirical behavior and robustness are examined in Section 3.1 and Section 3.7, while Section 4.4 clarifies that it has not been externally validated against independent load measures.

The eye-movement features differ in their response direction to visual load and in their dimensions. To unify direction and scale, each WESF feature was direction-aligned so that larger values correspond to higher visual load and then min–max normalized within the training folds, with the extrema estimated only from the training-fold windows and a small positive constant guarding against a zero denominator. Held-out feature values falling outside the training-fold range were not clipped; such values occurred in 0.049% of held-out windows (12/24,437) and were used as is (Appendix A).

After normalization, the WESF features no longer differ in dimension, yet they still differ in dispersion and distributional shape, and direct equal-weight aggregation could allow features with larger fluctuations to exert excessive influence. To mitigate this, objective weights for the WESF features were estimated within the training folds using the standard entropy-weight procedure [47], in which the information entropy of each normalized feature is computed over the training-fold windows and converted into a weight proportional to one minus the entropy. The entropy weight reflects only the distributional dispersion of a feature within the training-fold sample and does not indicate stronger load relevance, causality, or theoretical priority.

The entropy-weight method assigns weights solely according to the dispersion of an individual indicator within the training fold, without accounting for correlation and information conflict among indicators. To exploit both contrast intensity and inter-indicator conflict, a second set of objective weights was computed within each training fold using the criteria importance through the intercriteria correlation (CRITIC) method [48], in which each feature’s information content combines its standard deviation with its correlations to the remaining features. The two sets of weights were then fused into a unified weight through game-theory-based combination weighting [49], which solves for the combination coefficients by minimizing the total deviation between the combined weight and the two sets of base weights, followed by normalization. Using these fold-specific combined weights, the continuous VLI value for the *i*-th window in fold *f* was calculated as the weighted sum of the direction-aligned and normalized WESF features, as expressed in Equation (4).

(4) $${\mathrm{VLI}}_{i}^{\left(f\right)}=\sum_{j=1}^{J}w_{j}^{*,(f)}z_{ij}^{\left(f\right)}$$

where ${\mathrm{VLI}}_{i}^{\left(f\right)}$ is the continuous visual load index of the *i*-th window; $\sum_{j=1}^{J}w_{j}^{*,(f)}$ is the combined weight; and $z_{ij}^{\left(f\right)}$ is the direction-unified normalized feature.

To construct the classification target, the continuous VLI was discretized into three levels (low, medium, and high) according to the quantiles of the training-fold VLI distribution. The discretization thresholds are defined in Equation (5).

(5) $$\begin{array}{l} \tau_{1}^{\left(f\right)}=Q_{0.33}\left(\left\{{\mathrm{VLI}}_{i}^{\left(f\right)}|i\in W_{\mathrm{tr}}^{\left(f\right)}\right\}\right),\;\\ \tau_{2}^{\left(f\right)}=Q_{0.67}\left(\left\{{\mathrm{VLI}}_{i}^{\left(f\right)}|i\in W_{\mathrm{tr}}^{\left(f\right)}\right\}\right) \end{array}$$

where $\tau_{1}^{\left(f\right)}$ and $\tau_{2}^{\left(f\right)}$ denote the lower and upper thresholds obtained from the one-third and two-thirds quantiles of the training-fold VLI distribution.

(6) $$y_{i}^{\left(f\right)}=\left\{\begin{array}{ll} \mathrm{Low}, & {\mathrm{VLI}}_{i}^{\left(f\right)}\leq \tau_{1}^{\left(f\right)},\\ \mathrm{Medium}, & \tau_{1}^{\left(f\right)}<{\mathrm{VLI}}_{i}^{\left(f\right)}\leq \tau_{2}^{\left(f\right)},\\ \mathrm{High}, & {\mathrm{VLI}}_{i}^{\left(f\right)}>\tau_{2}^{\left(f\right)}. \end{array}\right.$$

where $y_{i}^{\left(f\right)}$ denotes the categorical VLI label of the *i*-th window in fold *f*, taking the values low, medium, or high according to the quantile thresholds of Equation (5).

As shown in Table 4, the distribution of combined weights across features is relatively balanced, with no single feature dominating label construction: the mean pupil diameter and the saccade-time-structure features carry higher weights, whereas the SD and coefficient of variation of pupil diameter carry lower weights. The entropy-weight and CRITIC methods differ markedly in the weights they assign to certain features, and the game-theory-based combination weighting compromises between the two, reducing the potential influence of any single weighting criterion on the VLI coordinate.

### 2.4. Vehicle Predictive Model and Input Constraints

This study imposes a strict modality-isolation constraint at the input level: all eye-movement statistics are used only to construct the VLI proxy label and never serve as input to any primary predictor. This constraint ensures that label construction and model prediction do not share the eye-movement data source, thereby ruling out, by design, any circular reasoning between the two.

Let the vehicle-kinematic feature sequence of the *i*-th window be denoted *X*_i,veh_, and the driving-style embedding of driver *d* be denoted $e_{\left(f)g(d\right)}$, where *g*(*d*) is the style cluster to which driver *d* belongs and the superscript (*f*) indicates that this embedding is estimated only from the training drivers of the *f*-th fold; the road type corresponding to the window is denoted *R*_i_. On this basis, five input settings (numbered M0–M4;see Section 2.5) are defined: M1 (Vehicle-only) takes only *X*_i,veh_ as input; M2 (Vehicle+Style) takes [*X*_i,veh_, *e*_(f)g(d)_] as input; and M0, M3, and M4 are road-control settings that take *R*_i_, [*R*_i_, *X*_i,veh_], and [*R*_i_, *X*_i,veh_, *e*_(f)g(d)_] as input, respectively. The five settings form a progressive contrast that separately quantifies the contributions of vehicle-kinematic information, the driving-style prior, and road-type confounding to the reconstructability of the VLI label. In addition, a Style-only probe, which takes only the within-fold driving-style embedding as input, is evaluated under the same protocol; it serves solely as a diagnostic reference for the individual-prior pathway and is not counted among the M0–M4 settings.

The modeling objective is not to select the optimal predictor but to ensure that comparisons across input sources are attributable; to this end, all primary analyses use the same low-capacity regularized logistic regression, with the M0–M4 settings sharing an identical model configuration. Four higher-capacity models—random forest, XGBoost, GRU, and Transformer-GRU—are used only for the model-invariance check. All predictors follow the same input boundary, and the model and hyperparameters are selected only within the training folds—for RF and XGBoost via nested driver-grouped cross-validation (Table A9)—with the exception of the pre-specified number of style clusters (*K* = 3; Section 2.5.2), so the held-out drivers do not participate in any model-selection step.

At the implementation level, the primary predictive models M1 and M2 concatenate the vehicle statistical features with the optional style embedding and feed them into the same classifier. To avoid driver-level information leakage, the parameters and cluster centers of the driving-style prior are estimated only within the training folds, and the held-out drivers are assigned according to the training-fold cluster centers (see Section 2.5.2 for details). In addition, this study retains a same-source probe model based on eye-movement features, which serves only as a positive control for diagnostic analysis and is not included in the main results, the main model-architecture diagram, or the main performance-comparison tables.

### 2.5. Input Configurations and Driving-Style Prior

The five input settings (M0–M4) are summarized in Table 5 according to their interpretive roles: M1 and M2 are the primary predictive models, testing the vehicle modality itself and the increment from the driving-style prior, whereas M0, M3, and M4 serve as road-confounding control and sensitivity analysis.

None of the five input settings contains any eye-movement input, consistent with the modality-isolation constraint of Section 2.4. Contrasting the primary models M1 and M2 with the road-control settings M0, M3, and M4 identifies, step by step, the increments of vehicle kinematics and the style prior beyond the road proxy, and the road-confounding diagnosis of Section 3.5 is conducted under the same model configuration.

#### 2.5.1. Vehicle-Kinematic Control Features

The vehicle-kinematic control features characterize a driver’s longitudinal and lateral control behavior within a window and serve as the basic descriptive quantities for constructing the driving-style prior. Specifically, under the unified 1 Hz time axis and 10 s window, five window-level statistics—the mean, standard deviation, maximum, minimum, and median—were computed for basic control quantities such as speed, longitudinal and lateral acceleration, jerk, and angular velocity; the symbol, definition, and unit of each feature are consistent with Table 3.

These window-level control features describe only vehicle kinematics and contain no road-type proportion or road-exposure information. To obtain a driver-level style prior, the control features of all windows of the same driver were aggregated at the driver level into a style vector; the same feature set also serves as the vehicle input for M1 (Vehicle-only) and M2 (Vehicle+Style). Because this aggregation uses all retained windows of a driver, including windows recorded after any given prediction time, the style prior is an offline, driver-level descriptor obtained in a transductive setting; online deployment to a new driver would instead require estimating the style vector from a preceding calibration period, which was not evaluated here. This caveat applies in particular to the future-state prediction task of Section 2.7 and Section 3.6.

#### 2.5.2. K-Means Driving-Style Extraction and Cluster Selection

The driving-style prior was obtained through unsupervised clustering of driver-level vehicle-control features: in the standardized feature space, the K-means algorithm [50] was used to cluster the driver-level control features. The number of clusters *K* was determined by jointly considering internal-validity diagnostics—the elbow method, the mean silhouette coefficient, the Davies–Bouldin index, and the Calinski–Harabasz index—and human-factors interpretability; the diagnostic results for candidate cluster numbers *K* = 2–6 are given in Section 3.2. These diagnostics were computed descriptively on the full 33-driver sample; *K* = 3 was therefore treated as a setting fixed before the modeling analysis rather than as a within-fold hyperparameter, whereas the cluster centers themselves were re-estimated within each training fold (Section 2.4).

To reduce the sensitivity of the clustering result to the initial centers, K-means clustering used multiple random initializations and selected the solution with the smallest within-cluster sum of squares as the final result. Clustering was performed in two ways: the clustering for description and visualization was based on all valid drivers, whereas the clustering for the M2 predictive model re-estimated the cluster centers within each training fold to avoid test-fold information leakage.

This study defines driving style as a driver-level statistical prior of vehicle kinematics, rather than a label of driving skill, safety level, or personality. Because model evaluation uses the driver as the held-out unit, the style prior must also be constructed at the driver level, so as to remain consistent with the driver-independent validation strategy (Section 2.1.5). For each driver, the window-level control statistics were aggregated into a driver-level style vector and standardized using the mean and standard deviation estimated only from the training-fold drivers; a held-out driver was then assigned to the nearest training-fold cluster center in the standardized feature space.

For the segment-referenced sensitivity analysis, each window-level control feature was standardized relative to the spatial segment containing the window midpoint, as specified in Equation (7), to account for fixed between-segment differences.

(7) $$z_{w,j}=\frac{x_{w,j}-\mu_{s(w),j}^{train}}{\sigma_{s(w),j}^{train}}s(w)\sigma^{train}$$

where $x_{w,j}$ and $z_{w,j}$ are the original and standardized values of feature *j* in window w, respectively; s(w) denotes the segment containing the window midpoint; and $\mu^{train}$ and $\sigma^{train}$ are the corresponding training-fold mean and standard deviation.

### 2.6. Vehicle Sequence Encoder and Training Objective

The vehicle sequence encoder plays a dual role: it is the primary model that generates the probability stream in the future high-VLI state prediction task of Section 2.7, and it acts as one member of the model-invariance check, while the primary analysis of same-window reconstruction remains the regularized logistic regression of Section 2.4. Structurally, it maps the vehicle-kinematic window sequence into a latent space through a projection layer, models global dependencies with Transformer attention, and preserves gated memory over time with a GRU; in the M2 setting the within-fold driving-style embedding is incorporated into the classification head. In accordance with Section 2.4, eye-movement features do not enter any predictor.

To address class imbalance among VLI categories, a weighted cross-entropy loss was applied during model training. The performance increments of different input configurations were quantified as defined in Equation (8).

(8) $$\begin{array}{l} \Delta \Phi_{\mathrm{style}}=\Phi (M2)-\Phi (M1),\\ \Delta \Phi_{\mathrm{veh}|\;\mathrm{road}}=\Phi (M3)-\Phi (M0),\\ \Delta \Phi_{\mathrm{style}|\;\mathrm{road}+\mathrm{veh}}=\Phi (M4)-\Phi (M3) \end{array}$$

where $\Delta \Phi_{\mathrm{style}}$, $\Delta \Phi_{\mathrm{veh}|\;\mathrm{road}}$, and $\Delta \Phi_{\mathrm{style}|\;\mathrm{road}+\mathrm{veh}}$ denote the performance increments introduced by style prior, vehicle features after road control, and style prior after adding vehicle features, respectively; and Φ(⋅) represents the selected high-VLI identification metric under each model setting.

Algorithm 1 presents the main training and evaluation procedure. The purpose of this algorithm is not to present additional results but to clarify, in a reproducible manner, the information boundary between the training and test folds. The training configuration of each candidate model and its role in this study are described below.
**Algorithm 1.** Driver-independent procedure for WESF→VLI label construction, reconstruction, and input-modality diagnosis**Input:** Window-level WESF, vehicle-kinematic, and road-type features of all drivers; folds *F* = 5; style clusters *K* = 3; bootstrap replications *B* = 200 **Output:** Out-of-fold performance of M0–M4; increments ΔΦ with driver-level 95% CIs 1: Partition all drivers into *F* disjoint folds, with the driver as the partition unit 2: **for** *f* = 1 **to** *F* **do**  3: Training folds only: normalize the WESF features, estimate the entropy–CRITIC combined weights, and construct the VLI label with quantile thresholds (Equations (4)–(6))  4: Training folds only: cluster the driver-level style vectors by K-means (*K* = 3)  5: Train the M0–M4 predictors (Table 5) on training-fold windows, with no eye-movement feature entering any predictor  6: Freeze all parameters; on held-out drivers, construct evaluation labels, assign the nearest style cluster, predict, and compute out-of-fold metrics 7: **end for** 8: Aggregate metrics across folds; compute the increments ΔΦ (Equation (8)) with driver-level bootstrap 95% CIs (*B* = 200) 9: **return** out-of-fold performance, increments, and diagnostic results (positive/negative controls; within-road stratification and relabeling)

Vehicle-Transformer-GRU takes the vehicle-kinematic window sequence as input and serves as the primary model for future-state prediction in Section 2.7, while also acting as a member of the model-invariance check; Vehicle+Style-Transformer-GRU builds on it by introducing a style embedding and a feature-fusion module to examine the style increment in the invariance check. Random forest (RF), XGBoost, and regularized logistic regression take the M1 or M2 feature groups as input, among which the regularized logistic regression is the primary model for same-window reconstruction, whereas RF and XGBoost are used only for the invariance check. The road-control probes correspond to M0, M3, and M4 and serve as road-confounding control and sensitivity analysis. This division is consistent with the fixed-model principle of Section 2.4, so that performance differences can be attributed unambiguously to changes in input source. The admitted features of the primary predictive model are restricted to vehicle-kinematic window statistics; the driving-style embedding is enabled only in M2 and M4, the road type enters only the road-control models, and eye-movement features are explicitly excluded from all predictors.

The evaluation system comprises six metrics: accuracy, Macro-F1, high-VLI recall, AUC, AP, and the increment ΔΦ defined in Equation (8). Accuracy reflects the overall proportion of correct classifications, but missing high-VLI windows weakens a model’s ability to capture the high-value region of the proxy space, so high-VLI recall serves as one of the primary model-selection metrics and Macro-F1 is reported to account for class imbalance. For the high-VLI binary probability stream, the area under the ROC curve (AUC) [51] and the average precision (AP, computed with high VLI as the positive class) [52] characterize ranking ability; both are threshold-independent but do not reflect probability calibration, and the thresholded classification behavior is reflected jointly by the accuracy and high-VLI recall reported in Section 3.2. For inference on model increments, the 33 drivers, rather than the 24,437 overlapping windows, constituted the independent sampling units (effective sample size *n* = 33). Accordingly, 95% confidence intervals for ΔΦ were constructed using driver-level bootstrap resampling [53] (B = 200) under the fixed-model configuration of Section 2.4. The null hypothesis was that adding the style prior did not change high-VLI identification performance after holding road control and the validation protocol constant. It was rejected at the 0.05 level when the corresponding confidence interval excluded zero. These driver-level bootstrap intervals were computed under the fixed-prediction configuration—resampling drivers over the fixed out-of-fold predictions—and quantify uncertainty conditional on the fitted within-fold models rather than population-level significance. Main-model AUC values are accordingly reported as point estimates, with interval estimates reserved for the pre-specified increment contrasts, and the appendix sensitivity analyses are reported as exploratory without adjustment for multiple comparisons. Intervals were taken as the 2.5th and 97.5th percentiles of the bootstrap distribution (percentile method); the complete random-seed registry and the fold composition are documented in Appendix A. Unless otherwise stated, all metrics were computed on the driver-independent held-out folds and reported as fold means.

For the threshold-based operating-point analysis introduced in Section 3.2, we additionally report the precision, recall, and window-level false-positive rate. To characterize the practical alarm burden, the window-level FPR is further converted into the number of false-positive windows per hour via Equation (9).

(9) $$FPW/h=FPR(1-\pi )\frac{3600}{\Delta }$$

where π is the high-VLI prevalence and Δ is the window step in seconds. For π = 0.333 and Δ = 5 s, FPW/h≈480×FPR. FPW/h counts false-positive windows rather than merged alarm events.

### 2.7. Future High-VLI Prediction and Persistence Baseline

Because the VLI is defined from within-window eye-movement statistics, same-window reconstruction does not test prospective temporal prediction; the same-source probe serves only as an upper-bound diagnostic.

To address this limitation, we formulated a driver-independent short-term high-VLI state estimation and prediction task, in which only non-gaze information from the current and preceding windows is used to estimate the binary high-VLI state of a subsequent target window. Because the eye-movement features are used exclusively to construct the target label and are excluded from the model inputs, this design blocks same-source circularity and allows the temporal contributions of the GRU’s gated memory and the Transformer’s attention mechanism to be assessed in a leakage-free setting.

For the *k*-th window of driver *d*, the prediction target is the binary high/non-high VLI label of the subsequent (*k* + *h*)-th target window, where *h* is the prediction horizon counted in window steps (5 s), with *h* = 1–4 corresponding to prediction horizons of 5, 10, 15, and 20 s, respectively. The model input uses only the non-ocular information up to the *k*-th window, that is, the vehicle-kinematic sequence or its combination with the training-fold driving-style prior.

Under the primary 50%-overlapping window scheme, at h = 1 (5 s), the target window overlaps the most recent input window by 5 s, corresponding to 50% of its duration. The 5 s horizon is therefore interpreted as a partially overlapping short-term state estimate (nowcast), rather than a fully prospective prediction. At h ≥ 2 (≥10 s), the input and target windows do not overlap, and the predictions are strictly prospective. Accordingly, the prospective conclusions are based on horizons of 10 s or longer. The analysis was also repeated using strictly non-overlapping 10 s windows, with the results reported in Section 3.6 and Table A5.

Consistent with the modality-isolation constraint of Section 2.4, eye-movement features are used only to construct the VLI label within the training folds and do not enter any future-prediction model; the setting that retains an eye-movement sequence input serves only as a same-source positive control and is not counted in the main results. Within-window VLI construction and the quantile thresholds are still estimated only within the training folds and applied to future windows, and the evaluation follows the driver-grouped validation protocol. The formal definitions of the future high-VLI target and the historical input sequence are given in Equations (10) and (11), respectively.

(10) $$y_{d,k}^{\left(h\right)}=I\left[y_{d,k+h}^{\left(f\right)}=\mathrm{High}\right],\;h\in \{1,2,3,4\}$$

where $y_{d,k}^{\left(h\right)}$ is the binary high-VLI target of the subsequent (*k* + *h*)-th window of driver *d*, taking the value 1 when the target window belongs to the high-VLI class and 0 otherwise; and *h* is the prediction horizon counted in window steps.

(11) $$S_{d,k}^{\left(L\right)}=(u_{d,k-L+1},u_{d,k-L+2},\ldots ,u_{d,k}),\;{\hat{p}}_{d,k+h}^{\left(L\right)}=F_{\theta }(S_{d,k}^{\left(L\right)})$$

where $S_{d,k}^{\left(L\right)}$ denotes the historical input sequence of driver *d* up to window *k* with a look-back length of *L*; $u_{d,k}$ represents the input feature vector at window *k*; ${\hat{p}}_{d,k+h}^{\left(L\right)}$ denotes the predicted probability of high VLI at target window *k* + *h*; and $F_{\theta }(\cdot )$ represents the prediction model.

To define the reference level of the future-prediction task, this study introduces a persistence baseline: the current high/non-high VLI state of the *k*-th window at the prediction origin is used directly as the prediction for the future (*k*+*h*)-th window. Because this baseline uses the current window’s label information (derived from eye-movement statistics), it does not constitute a deployable predictive model and serves only as a reference upper bound determined by state autocorrelation; the decay of its performance with the prediction horizon reflects the dissipation rate of state-autocorrelation information. The temporal-structure gain of the vehicle sequence model is accordingly evaluated in terms of its stable gain over the shallow vehicle probe and its more gradual temporal decay relative to this baseline, with the benchmark results given in Section 3.6.

## 3. Results

### 3.1. VLI Label Distribution and Robustness

To examine the soundness of the VLI label construction, Table 6 summarizes the composition of the low-, medium-, and high-class label proportions under training-fold quantile discretization, the difference in central tendency of the VLI between road types, and the heterogeneity of driver-level high-VLI proportions.

In the label-robustness check, the VLI label was regenerated within the training folds using four alternative weighting schemes—entropy weighting, CRITIC, equal weighting, and the first principal component (PCA-1). Table 7 reports the agreement rate between each alternative label and the primary VLI and compares the predictive stability under the M1 (Vehicle-only) and M2 (Vehicle+Style) input settings; because the pupil features are susceptible to ambient-illumination interference, Table 7 also includes a VLI variant with all pupil features removed.

Table 6 shows that training-fold quantile discretization produced approximately balanced class proportions. The proportion of high-VLI windows was 38.0% on Road A and 27.3% on Road B, while the corresponding driver-level proportions ranged from 5.2% to 89.1%. These road- and driver-level differences motivated the subsequent road-confounding analyses and driver-grouped evaluation.

As shown in Table 7, the agreement rate between the four alternative weightings and the primary VLI is no lower than 87.6%; across the variants, the AUC of M1 remains stable within the range of 0.562–0.617, and the direction of the style increment of M2 is consistently positive, indicating that both the label and the conclusions are insensitive to the choice of weighting scheme. After removing all pupil features, the agreement rate drops to 73.3%, yet the AUC of M1 still reaches 0.562, close to the primary-label setting, showing that the main conclusions do not depend on the illumination-susceptible pupil channel.

### 3.2. Same-Window Reconstruction from Vehicle and Style Inputs

This section follows the fixed model configuration of Section 2.4, using regularized logistic regression to benchmark each of the M0–M4 input settings, so that performance differences are attributable solely to the input source rather than to model complexity.

The out-of-fold primary metrics for each input setting and the increment test after introducing the style prior are presented in Table 8. A driver-by-driver review of the out-of-fold discriminative performance of M1 and M2 shows that the AUC of the vast majority of drivers lies above chance level and that most improve after the style prior is added; for a few drivers, however, the AUC is markedly lower, echoing the driver-level heterogeneity of high-VLI proportions between 5.2% and 89.1% shown in Table 6.

As Table 8 shows, M1 achieves an AUC of 0.589 on held-out drivers, higher than the 0.562 of M0 and the 0.532 of Style-only, indicating that the vehicle-kinematic window features have a reconstruction ability that exceeds both the road proxy and a purely individual prior, although the absolute level is limited, consistent with the expectation that the vehicle is only a weak proxy. Adding the style prior raises the AUC of M2 to 0.633 and high-VLI recall from 0.109 to 0.143, although out-of-fold classification at the 0.50 threshold remains conservative and biased toward suppressing window-level false positives; the style increment ΔAUC of 0.044 has a driver-level 95% confidence interval [−0.005, 0.113] that includes zero and is therefore interpreted only in an exploratory sense. The high-VLI recall of M0 is 0.000, meaning that road type alone cannot identify any high-VLI window, and M4 attains the highest AUC (0.642) but serves only for sensitivity analysis.

To complement the threshold-independent AUC results, Table 9 further reports the precision, recall, false-positive rate (FPR), and false-positive windows per hour (FPW/h) across groups under four threshold selection strategies. It should be noted that all data-driven thresholds were determined exclusively on the training group to avoid information leakage during evaluation.

Table 9 also reveals the trade-off between detection capability and window-level false-positive burden. At the default threshold of 0.50, both M1 and M2 behaved conservatively, yielding recalls of 0.109 and 0.143, with 29 and 30 false-positive windows per hour (FPW/h), respectively. When the target recall was raised to at least 0.70, both models detected substantially more high-VLI windows, but at the cost of increased false-positive windows, with FPW/h rising to 290 and 259, respectively. Overall, at the Max-F1 and recall-constrained operating points, M2 achieved higher precision together with lower FPR and FPW/h while maintaining a slightly higher recall, indicating a more favorable balance between detection capability and window-level false-positive burden.

**Figure 4:**
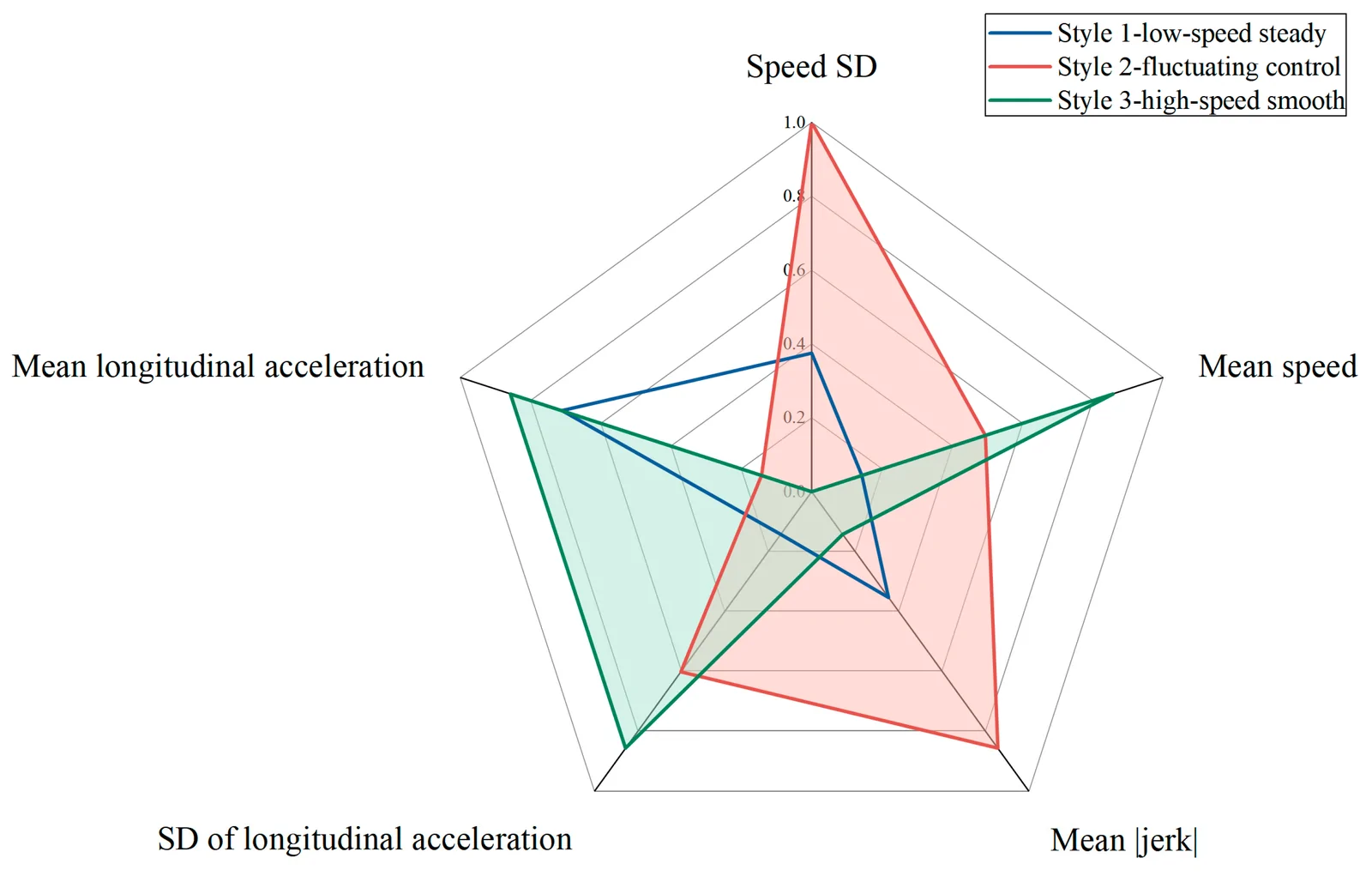
Control-statistics profiles of the three style-cluster centers.

To construct the driver-level style prior, this study used only vehicle-kinematic control statistics and re-estimated the cluster centers per training fold in the predictive model, with road type not entering the style vector. Across candidate cluster numbers *K* = 2–6, the mean silhouette coefficient peaked at *K* = 3 (0.450) and the decline in the within-cluster sum of squares leveled off thereafter, showing a clear elbow pattern; with a sample of only 33 drivers, a larger *K* would produce style clusters too small for stable interpretation. Therefore, taking internal-validity indices and human-factors interpretability together, *K* = 3 was selected, and the standardized center features of each style cluster and the driver distribution are shown in Figure 4 and Table 10.

As shown in Table 10, the three style clusters contain thirteen, fourteen, and six drivers, respectively, and differ mainly in mean speed and mean absolute jerk: Style 1 has the lowest mean speed and intermediate jerk; Style 2 has intermediate speed but the highest mean absolute jerk, reflecting larger longitudinal-control fluctuation; and Style 3 has the highest speed and the lowest jerk. The style labels are used solely as descriptive shorthand for these within-sample kinematic profiles rather than as validated driving-style categories; given the 33-driver sample and cluster sizes of thirteen, fourteen, and six, the cluster number, centers, and membership should be regarded as dataset-specific observations, whose stability and generalizability to other driver populations, routes, and traffic conditions remain to be established. Although the clusters are unequal in size, each satisfies the pre-specified minimum cluster-size criterion of Section 2.5.2; the cross-cluster standardized values further confirm that the separation is most pronounced along the speed and jerk dimensions.

The clustering results above describe the vehicle-control differences in the current sample and provide the driver-level prior for M2 according to the within-fold estimation and assignment rules of Section 2.5.2. To establish reference bounds for the input-source diagnosis, two controls were evaluated under the same protocol: a random-representation negative control, repeated 100 times with independent draws, yielded a mean AUC of 0.498 ± 0.063 (range 0.333–0.656; Appendix A), providing an empirical random-level reference, whereas a same-source eye-movement probe reached an AUC of 0.997, showing that once eye-movement features are allowed into the predictive input, same-source circularity emerges and defines the same-source upper bound. The performance of M1 and M2 lies between these two bounds and represents a cross-source predictive association obtained, under the evaluated controls, without any access to eye-movement information.

### 3.3. Model-Invariance Check

The model-invariance check confirms that the input-source conclusions do not depend on a specific model architecture. Under the M1 and M2 inputs, RF, XGBoost, GRU, and Transformer-GRU are compared with the main-analysis logistic regression in Table 11, all following the modality-isolation constraint of Section 2.4; the increment after road control is tested separately in Section 3.5 and lies outside this table. Across the five architectures, the increment of M2 over M1 is positive for AUC and high-VLI recall, and positive in direction for accuracy, Macro-F1, and AP, though the AUC increment is generally small and architecture-dependent (ΔAUC 0.008–0.044, with RF almost flat).

Table 11 further shows that, under the M1 input, the AUC of the five architectures ranges from 0.570 to 0.624, and under M2 from 0.587 to 0.642. The high-capacity sequence models do not markedly surpass the regularized logistic regression on the same-window reconstruction task, supporting the use of a fixed low-capacity predictor for the main analysis. Across architectures, the gain from the style prior is positive in direction but limited in magnitude, consistent with the main results. The full metrics of the model-invariance check across all architectures are provided in Table A1 (Appendix A).

### 3.4. Interpretability Analysis via PDP and SHAP

After determining the two predictive inputs M1 and M2, partial dependence plots (PDPs) [54] and SHapley Additive exPlanations (SHAP) values were constructed on the fixed predictor used in the main analysis, in order to characterize the marginal response and local contribution of the vehicle-kinematic and driving-style features to the high-VLI probability.

**Figure 5:**
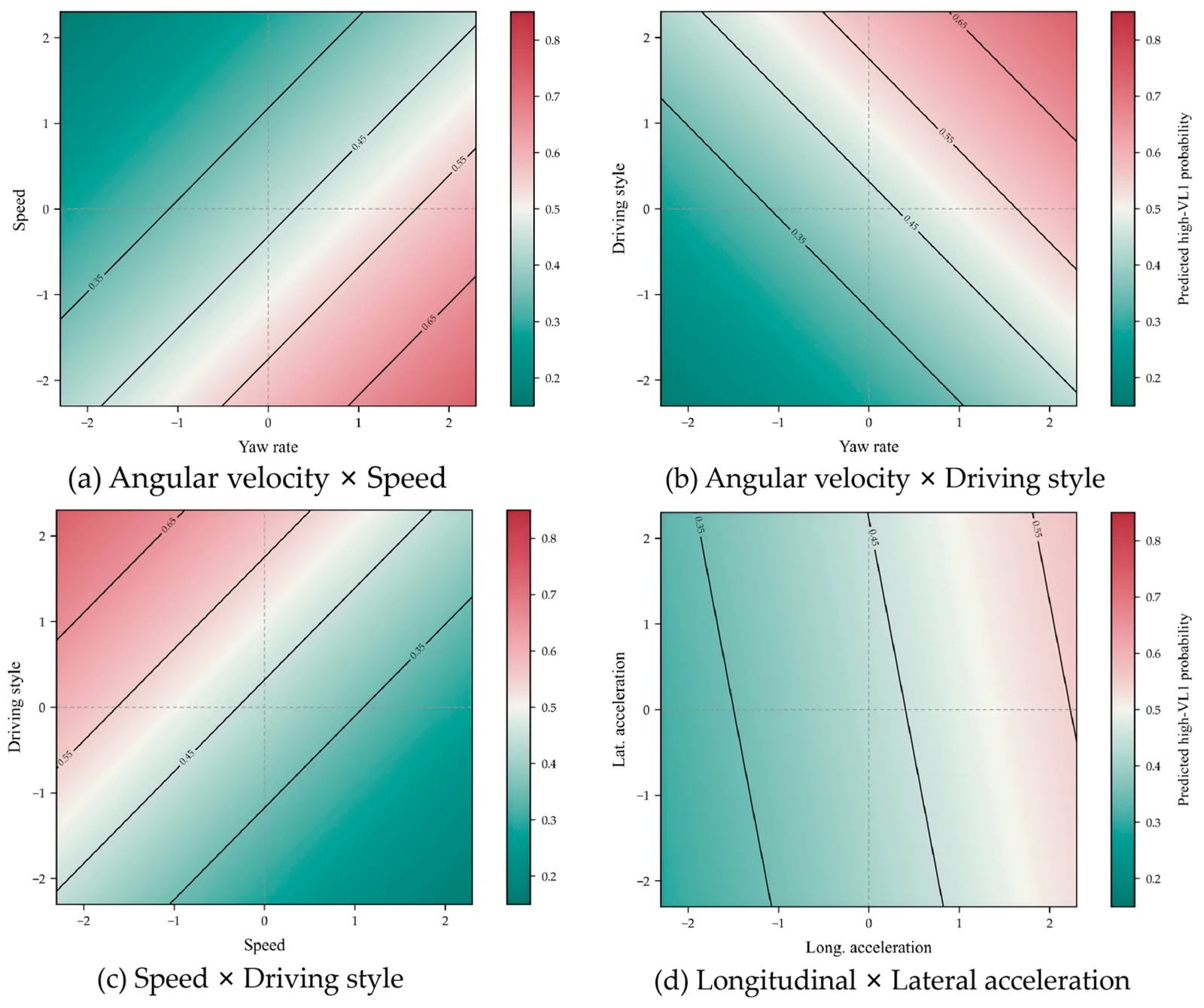
Bivariate high-VLI-probability PDP response surfaces: (**a**) angular velocity × speed; (**b**) angular velocity × driving style; (**c**) speed × driving style; (**d**) longitudinal × lateral acceleration.

The PDP and SHAP analyses are strictly restricted to the vehicle and style inputs and contain no eye-movement features used for label construction. The bivariate PDP response surface of the vehicle- and style-input combinations for the high-VLI probability is shown in Figure 5.

The importance ranking of the main vehicle response features is summarized in Table 12. The mean absolute SHAP proportion reflects the strength of association between the vehicle model’s predicted probability and the input variables.

The SHAP absolute-value proportion in Table 12 measures each feature’s contribution to the model’s predicted probability, which is not on the same scale as the out-of-fold discriminative gain in Table 8: a feature may occupy a moderate share in prediction attribution yet not bring a statistically significant ranking improvement for unseen drivers. The angular-velocity statistics rank first, consistent with the frequent steering and merging maneuvers on urban-expressway sections with dense entrances and exits, echoing Xu et al. [6]; the speed statistics rank second with a predominantly negative association, but being collinear with road type, their response partly reflects road-exposure differences and should be read together with the road-control results of Section 3.5 rather than interpreted causally. Driving style ranks third with an attribution share of 22.3%, which arises mainly from the strong heterogeneity of driver-level high-VLI proportions; the style prior shifts each driver’s high-VLI probability as a whole and thus gains a considerable attribution share, yet under driver-independent evaluation with style characterized by only three coarse clusters, this heterogeneity does not translate into significant window-level discrimination. The high attribution ranking and the statistically non-significant increment are therefore not contradictory—together they form a self-consistent manifestation of the same inter-driver heterogeneity under different metrics. For a representative driver, the window-level VLI trajectory and the corresponding Vehicle+Style out-of-fold probability are illustrated in Figure A2 (Appendix A).

**Figure A1:**
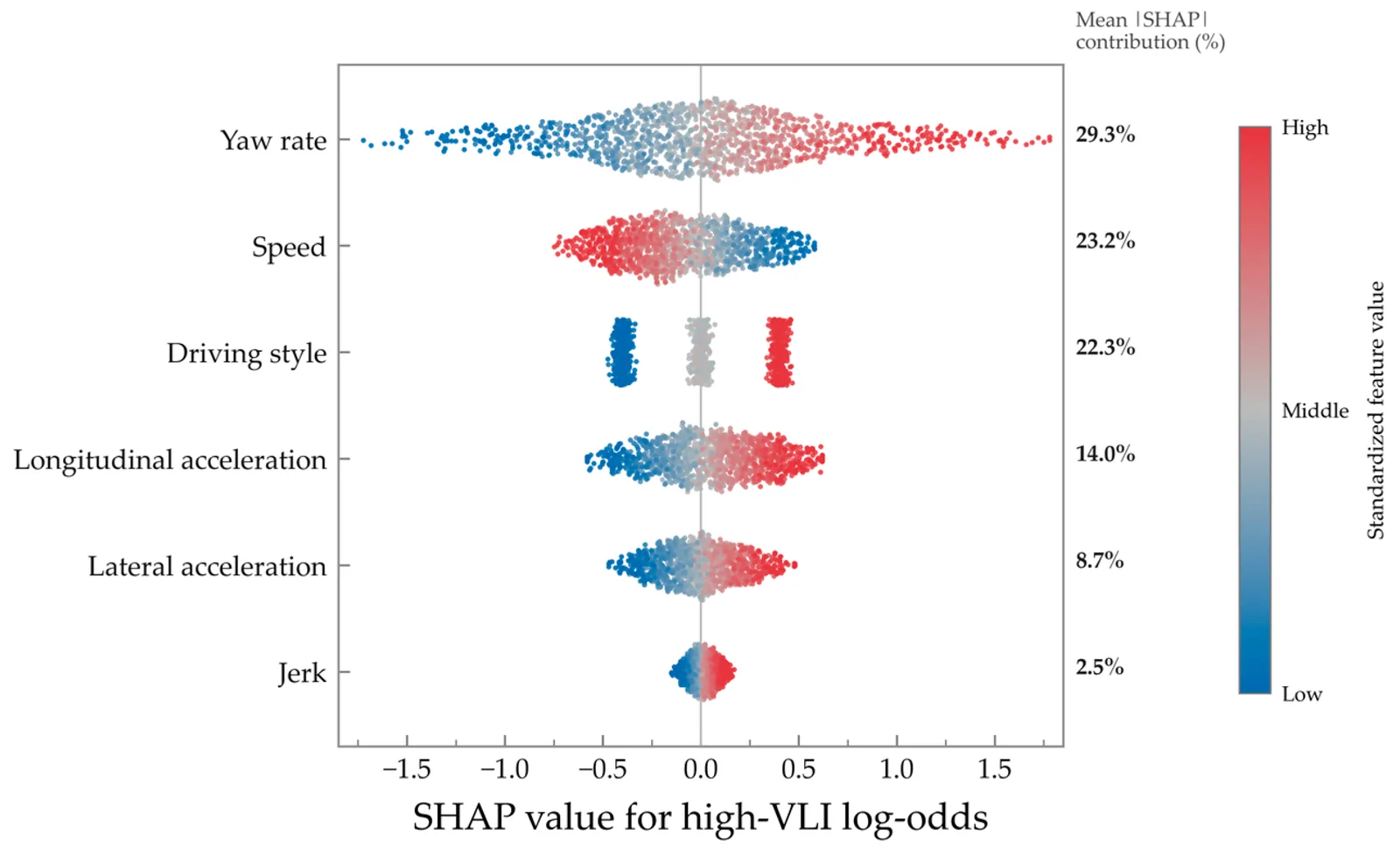
SHAP contributions of the vehicle-kinematic and driving-style features for high-VLI prediction.

**Figure A2:**
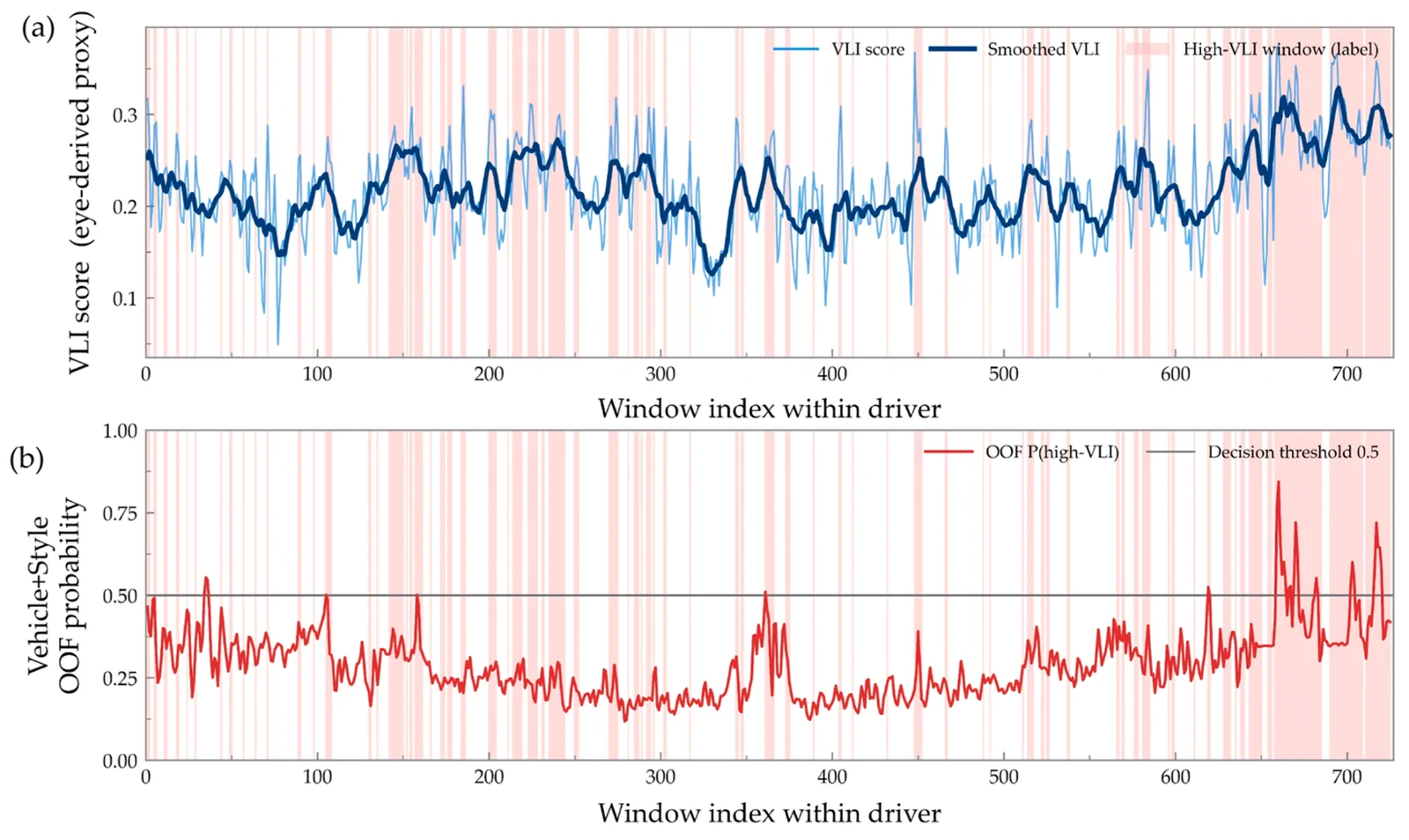
VLI trajectory and out-of-fold probability for a representative driver (Driver 18): (**a**) window-level eye-movement-derived VLI proxy; (**b**) Vehicle+Style out-of-fold probability of high VLI.

As shown in Figure 5, the first-order gradient directions of the high-VLI probability across the four bivariate response surfaces are consistent with the directions of influence reported in Table 12. In Figure 5a–c, the predicted probability generally increases with driving style and decreases with speed, and angular velocity exhibits a positive gradient in the surfaces it forms with speed and with driving style. In contrast, the surface formed by longitudinal and lateral acceleration in Figure 5d is the flattest, in line with the small attribution shares of these two features. Because the vehicle-kinematic input as a whole is only a weak proxy, with an M1 out-of-fold AUC of 0.589, these gradients should be interpreted as marginal tendencies in probability ranking rather than as causal effects. The corresponding SHAP contribution distributions of the vehicle-kinematic and driving-style features are presented in Figure A1 of Appendix A.

### 3.5. Road-Confounding Control and Vehicle-Increment Test

The input-source diagnosis used low-capacity out-of-fold probes and maintained the same driver-grouped evaluation protocol as the main analysis, with the aim of determining whether the three non-ocular sources—vehicle, style, and road—each carry separable first-order discriminative information. Because road type may simultaneously affect the vehicle-kinematic variables and the VLI label distribution, road-confounding control forms an integral part of the main results; the out-of-fold performance of M0–M4 has already been incorporated into Table 8, and the role division is given in Table 5.

The diagnosis uses two increments as criteria: M2 relative to M1 tests whether the style prior provides information beyond the vehicle features, and M3 relative to M0 tests whether the vehicle’s predictive ability exceeds the road proxy. The vehicle and style increments are further examined under within-road stratification and a within-road relabeling setup. All ΔAUC values are accompanied by driver-level bootstrap 95% confidence intervals, and the increment-test results are listed in Table 13.

Under within-road stratification, the AUC of M0 drops to near chance level (0.512 for Road A and 0.501 for Road B), indicating that road-type information has almost no remaining discriminative ability within a road, whereas M1 and M2 maintain above-chance AUCs under the same setup (0.573/0.564 and 0.591/0.582, respectively), indicating that the discriminative information provided by the vehicle and style features was not driven solely by differences between the two road categories. The within-road relabeling results further show that, even after removing the between-road label-distribution difference, M1 and M2 still maintain global AUCs of 0.565 and 0.583, respectively; that is, the conclusion that the vehicle modality carries a stable signal beyond the road remains valid under this setup.

As shown in Table 13, the vehicle increment M3 − M0 after road control is 0.042, and its driver-level 95% confidence interval [0.006, 0.078] excludes zero, indicating that vehicle kinematics provide an increment distinguishable from zero under the conditional driver-level bootstrap; the style increment M4 − M3 after road control is 0.038, and its confidence interval includes zero, so it is interpreted only in an exploratory sense. Together, these two criteria show that the vehicle’s predictive ability cannot be explained by the binary road-type proxy alone, whereas the style prior maintains a consistent pattern that is positive in direction but does not reach statistical significance across the control settings.

To control for within-road confounding beyond what the binary road-type variable can capture, an additional segment-level control analysis was performed. The fixed test route was discretized into 14 spatial segments delimited by interchanges, with six segments on Road A and eight on Road B and a mean segment length of 1.7 km. In the control settings M0′ and M3′, a one-hot-encoded segment identifier replaced the binary road-type input. Because the segment indicators account for time-invariant differences across the predefined segments, including differences associated with curvature, lane configuration, ramp density, and local speed limit, this specification provides a more stringent control for segment-level spatial heterogeneity than the binary road-type indicator. However, it does not control for geometric variation within each segment or time-varying traffic conditions. As shown in Table A2, adding the vehicle features under this setting increased the AUC from 0.581 to 0.615, corresponding to a ΔAUC of +0.034 and a driver-level bootstrap 95% confidence interval of [0.003, 0.065]. Although this increment was attenuated relative to that under the binary road-type control, its confidence interval still excluded zero. These results indicate that the vehicle features retain incremental predictive information after accounting for the between-segment fixed differences captured by the segment indicators.

Table A3a summarizes the spatial composition of the analysis windows. Ramp-influenced and connecting-ramp windows exhibit the highest mean VLI (0.246 and 0.252, respectively) and the largest angular-velocity magnitudes, together with the lowest mean speeds, consistent with previous findings on interchange areas [6,7,8] and with the SHAP attribution in Section 3.4. The main results were insensitive to this classification (Table A3b): excluding boundary-crossing and connecting-ramp windows, or recoding road type as {A, B, Ramp}, left all increments essentially unchanged, and within the basic-segment subsample alone the vehicle increment remained positive with a confidence interval excluding zero (ΔAUC = +0.036, 95% CI [0.002, 0.070]).

Directional differences were likewise small, as detailed in Table A4. Under the joint road-by-direction control, the vehicle increment was +0.040, with a driver-level bootstrap 95% confidence interval of [0.005, 0.075], closely matching the primary estimate; the direction-stratified out-of-fold AUCs differed by less than 0.01 between the outbound and return runs. The descriptive indicators showed only minor directional differences: the high-VLI proportion was slightly higher outbound than on return, at 38.9% versus 37.1% on Road A.

### 3.6. Benchmarking Future High-VLI Prediction

**Figure 6:**
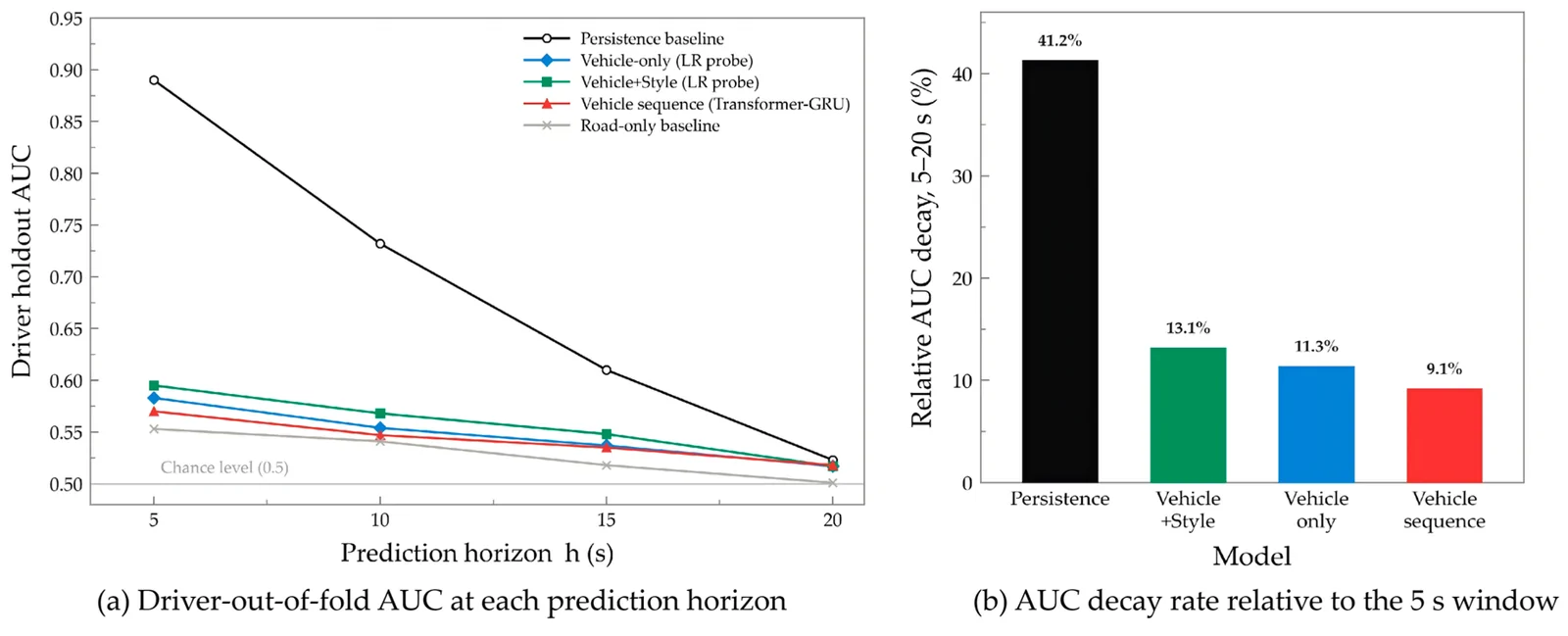
Temporal-decay characteristics of future high-VLI state prediction: (**a**) driver-out-of-fold AUC at each prediction horizon; (**b**) AUC decay rate relative to the 5 s window.

**Figure 7:**
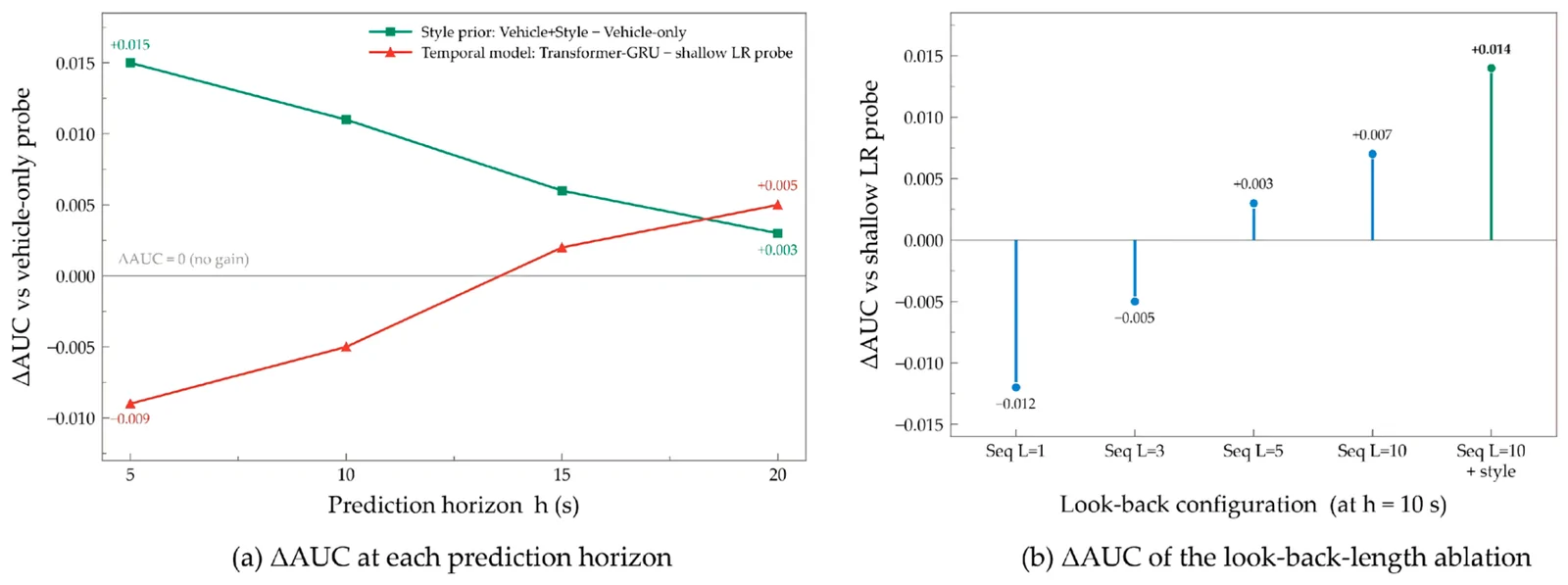
Increment analysis of future high-VLI state prediction: (**a**) ΔAUC at each prediction horizon; (**b**) ΔAUC of the look-back-length ablation.

To evaluate the predictability of future high-VLI states while excluding same-source circularity, Figure 6 compares the persistence baseline defined in Section 2.7, M0, M1, M2, and the vehicle sequence model in terms of driver-out-of-fold performance across prediction horizons of 5–20 s, and Figure 7 reports the corresponding ΔAUC increments.

As shown in Figure 6, the persistence baseline achieves an AUC of 0.891 at the 5 s horizon but falls to 0.524 at *h* = 20 s, indicating that the short-term autocorrelation information of the VLI state dissipates rapidly as the horizon lengthens. By contrast, the absolute AUCs of the vehicle-input models remain modest but decay more gradually with the horizon, with relative reductions of 9.1–13.1% from the 5 s level compared with 41.2% for the persistence baseline, as shown in Figure 6b, and essentially converge to the persistence baseline at *h* = 20 s. This pattern suggests that the information carried by vehicle kinematics and by state autocorrelation becomes comparable in magnitude at long horizons, although the available evidence remains insufficient to show that the former surpasses the latter. The AUC of M0 stays closest to chance level across all horizons, further confirming that road type alone has limited ability to predict future high-VLI states. Meanwhile, the high-VLI recall of the vehicle sequence model declines as the horizon lengthens, indicating that early warning of high-VLI states at long horizons remains challenging. For this prospective task, only descriptive comparisons of the direction and magnitude of each increment are reported, and no claims of statistical significance are made.

As a sensitivity analysis, we repeated the future-prediction task using strictly non-overlapping 10 s windows; the results are reported in Table A5. For horizons h ≥ 10 s, the results are consistent with those of the main configuration in both direction and magnitude. The persistence baseline degrades rapidly, with its AUC decreasing from 0.703 at h = 10 s to 0.505 at h = 30 s, and it is outperformed by the vehicle-feature-based models beyond h = 10 s. In contrast, the Vehicle-only and Vehicle+Style models exhibit a much more gradual decline; for example, the AUC of M2 decreases only from 0.588 to 0.552 over the same range.

To further examine the effect of the amount of usable historical information on prediction performance, an ablation experiment was conducted with the look-back length *L* as the variable, and the results are summarized in Figure 7b. The metrics for each configuration are recomputed on the valid sample set corresponding to the respective look-back length and are used only for relative comparison among different values of *L*; they are not directly comparable with the values under the benchmark-evaluation setup of Figure 6, and the difference at *h* = 10 s between the two arises precisely from the difference in sample sets. The shallow vehicle probe used as the reference is an out-of-fold logistic-regression probe that uses only the current-window features (*L* = 1).

As Figure 7b shows, as the look-back length *L* increases from 1 to 10, the AUC of the vehicle sequence model under the Vehicle-only input rises monotonically (0.567 to 0.578), its ΔAUC relative to the shallow vehicle probe turns from negative to positive, and adding the style prior at *L* = 10 attains the best result among the configurations tested, indicating that a longer historical sequence can provide a small but directionally consistent gain. The limited magnitude of the gain shows that, under the current feature setting and sample size, predictability is constrained mainly by the information content of the vehicle modality rather than by the look-back length within the tested range. Figure 7a further shows that the increment of M2 over M1 narrows progressively as the horizon lengthens, whereas the increment of the vehicle sequence model over the shallow probe turns from negative to positive with the horizon, and the two converge to the same order of magnitude at *h* = 20 s.

### 3.7. VLI Distribution and Model Performance by Window Category

Following the procedure described in Section 2.1.5, the analysis windows were divided into three categories: ramp-related windows, defined as the union of ramp-influenced and connecting-ramp windows and serving as a proxy for merge and diverge operations; lane-change/overtaking-related windows, identified by a kinematic trigger; and the remaining regular-driving windows. Consistent with the spatial classification in Section 2.1.5, the 87 boundary-crossing windows were excluded from the ramp-related category and entered the non-ramp pool. Within the non-ramp pool, these windows were assigned to either the lane-change/overtaking-related category or the regular-driving category using the joint kinematic trigger rule defined in Equation (1). To assess the reliability of this rule, 180 triggered windows were randomly sampled and verified against the forward dashcam video, yielding a verification precision of 87.8%. Exhaustive video-based manual maneuver annotation of all 24,437 windows was impractical and could itself introduce annotator variability. The kinematic trigger, supported by video verification of a random sample of triggered windows, was therefore used as a scalable and reproducible operational approximation. The exclusion analysis in Table A3b showed that the main results were insensitive to the treatment of the boundary-crossing windows.

As shown in Table 14, regular-driving windows accounted for 20,647 windows (84.5%), lane-change/overtaking-related windows for 1140 (4.7%), and ramp-related windows for 2650 (10.8%). The indicators exhibited a consistent gradient across the three categories: from regular driving through lane-change/overtaking-related to ramp-related windows, the mean VLI was 0.207, 0.238, and 0.247, the high-VLI proportion 31.3%, 42.0%, and 45.5%, and the mean predicted high-VLI probability of M2 0.31, 0.38, and 0.41, respectively. This monotonic gradient across maneuver categories matched the expected ordering of visual-demand intensity, from regular driving through lane-change/overtaking to ramp-related windows. It therefore provided descriptive known-groups consistency evidence for the VLI proxy, complementing the weighting-robustness results in Table 7. The within-category out-of-fold AUCs were 0.571, 0.596, and 0.603 for M1 and 0.612, 0.633, and 0.641 for M2, with M2 exceeding M1 in every category by 0.037–0.041. At the descriptive level, these results link the model outputs to identifiable traffic-operation contexts and are consistent with the SHAP attribution structure of Section 3.4; merge, diverge, lane-change, and overtaking windows typically involve more frequent lateral control adjustments, plausibly corresponding to higher visual-sampling demand and more pronounced angular-velocity activity.

It should be emphasized that these gradients are descriptive and should not be interpreted as causal effects of traffic operations on visual load. Given the unified 1 Hz time base, the classification identifies windows in which the corresponding operations are concentrated rather than individual maneuver events (Section 2.2); because inter-vehicle range data were not collected, car-following and free-flow driving cannot be reliably separated and are pooled into the regular-driving category. In addition, the within-category AUCs in Table 14 were computed from the pooled driver-grouped out-of-fold predictions rather than from separately trained category-specific models.

## 4. Discussion

### 4.1. Cross-Source Reconstructability and the Role of the Style Prior

Across held-out drivers, vehicle kinematics retained measurable information about the eye-movement-derived VLI proxy. Adding vehicle features to the binary road-type control model improved AUC by 0.042 (95% CI: 0.006–0.078), indicating measurable information beyond this coarse contextual proxy. Absolute discrimination nevertheless remained modest, positioning vehicle kinematics as a complementary rather than standalone information source. This finding is consistent with the conclusions of Xu et al. [6], Mu et al. [7], and Zhang et al. [8] on high-density entrance/exit sections: road geometry and merge–diverge configurations both shape the vehicle-kinematic response and raise the driver’s load level, thereby providing a plausible contextual explanation for cross-source prediction from the vehicle modality. The attribution structure of Section 3.4 supports this interpretation—the model uses angular velocity as a positive indicator and low speed as an accompanying feature, and this structure remains stable when same-source circularity is blocked, indicating that the load-related information carried by the vehicle modality shows a pattern consistent with contextual contributions rather than being a statistical artifact induced by label construction. The increment of the driving-style prior is consistently positive in direction but not statistically significant, and this pattern is stable across Section 3.2 and Section 3.5, the five architectures in Table 11, and the road-control settings in Table 13. As the attribution analysis of Section 3.4 shows, it reflects an overall shift in the driver-level high-VLI base rate: the coarse style clusters provide a driver-level prior in a sample with highly heterogeneous high-VLI proportions, but with only three clusters and 33 drivers this inter-driver information does not translate into a significant window-level discriminative gain, and a genuinely limited contribution of style to the window-level state cannot be excluded. Accordingly, the cluster structure itself is treated as a sample-specific summary rather than a general driving-style taxonomy. This is consistent with driving-style research [20,21,22,23,24,25,26] holding that style is a long-term stable individual attribute whose contribution to state-level prediction depends on finer representations and larger driver samples.

As shown in Table A6, the style increment remained positive after removing mean speed from the style representation and after applying the segment-referenced standardization defined in Equation (7), with ΔAUC values of +0.032 and +0.037, respectively. Both estimates were attenuated relative to the primary increment of +0.044, and their driver-level 95% confidence intervals included zero. These results suggest that road and traffic context partly contribute to the style prior, while its residual contribution remains exploratory.

In terms of cross-study comparison, most existing studies on driver-load prediction report performance under same-source settings—where the label and the input come from the same sensor, or where features from the label source are allowed into the model—and their values are not directly comparable with the cross-source, driver-independent setting of this study; to our knowledge, no publicly available benchmark is directly comparable under the strict setting of an eye-movement-derived label, purely vehicle inputs, and driver-grouped validation. The persistence baseline, the road-type baseline, and the same-source positive control therefore together form an internal reference frame within which the relative level of the vehicle modality can be located, and the 0.589 discriminative level should be regarded as a realistic estimate obtained under conservative conditions—its significance lies in accurately delineating the capability boundary of the vehicle modality rather than in providing a directly deployable high-precision classifier. Under this equally strict frame of reference, any performance change brought by richer vehicle features, larger driver samples, or multisource fusion in subsequent research can be measured fairly, avoiding apparent improvements obtained through same-source circularity or road-proxy effects; all the criteria, decision rules, and confidence intervals reported here provide a reproducible benchmark for such comparisons.

### 4.2. Road-Confounding Control and Circularity Diagnosis

The road-confounding diagnosis is the key design that distinguishes this study from existing naturalistic-driving-behavior research [27,28,29,30,31,32,33]. The within-road stratification and relabeling results reported in Section 3.5 show that the discriminative ability of M0 drops to near chance level within a road, whereas the vehicle and style models still remain above chance under the same setup; combined with the driver-level confidence interval for the M3 − M0 vehicle increment, these results suggest that the predictive contribution of the vehicle features cannot be explained solely by the binary road-type proxy. The additional segment-level analysis reported in Section 3.5 further reduced concerns regarding spatially fixed within-road confounding. Nevertheless, residual confounding associated with within-segment geometric variation and time-varying traffic conditions cannot be completely excluded, as acknowledged in Section 4.4.

More fundamentally, contextual factors such as traffic conditions, interchange geometry, maneuver demand, and surrounding vehicles should not be treated solely as nuisance confounders. These factors can jointly shape vehicle-kinematic responses and the eye-movement-derived VLI proxy, creating shared structure on which cross-source prediction may depend (Section 4.1). The control analyses were therefore designed to test whether the contribution of vehicle kinematics was reducible to coarse contextual descriptors or spatially fixed road characteristics. These controls included road type, segment identity, direction, and maneuver category (Section 3.5 and Section 3.7). They were not intended to eliminate all contextual influence, because doing so would also remove part of the phenomenon being modeled. Separating context-mediated from driver-state-mediated pathways would require additional sensing, such as leading-vehicle range data, and remains a priority for future work.

At the methodological level, the diagnostic scheme of this study directly targets the same-source circularity risk in proxy-label research: when the label is deterministically constructed from eye-movement features, a model containing eye-movement input may yield reconstruction performance that approaches the upper bound yet is uninformative, as exemplified by the same-source positive control reported in Section 3.2 (AUC = 0.997). By strictly confining eye movements to label construction within the training folds, using the driver as the leakage-control unit, and adding positive and negative controls together with road control, this scheme enables the distinction between high apparent reconstruction performance and cross-source predictive association under the evaluated controls, and it can be extended to load-sensing research based on physiological signals such as heart rate variability [7] or EEG [9].

Although the VLI lacks external criterion validation, three complementary lines of indirect evidence support its use as a research proxy. First, as noted in Section 2.2, each constituent feature family—pupillary, fixation, and saccadic—has independent literature support as a load-sensitive indicator [1,2,3,4,5]. Second, four alternative weighting schemes and a pupil-removed variant produced concordant labels and stable downstream conclusions, indicating construction robustness (Table 7). Third, VLI values increased monotonically across window categories ordered by expected maneuver demand, providing descriptive known-groups consistency evidence (Section 3.7). These indirect findings support the VLI as an operational proxy for hypothesis-generating research but do not constitute external validation. Validation against subjective scales or independent physiological measures remains necessary for deployment-oriented claims and is identified as future work in Section 4.4.

### 4.3. Capability Boundaries and Application Implications

This study further delineates the capability boundary of the vehicle modality in the future high-VLI state prediction task. Within the short horizon of 5–10 s, the persistence baseline maintains the highest AUC, reflecting the strong short-term autocorrelation of the load state; when the prediction horizon is extended to 20 s, the vehicle sequence model essentially converges to this baseline, but by then the absolute AUCs of both are already close to chance level; extending the look-back length and adding the style prior can bring a directionally consistent gain of limited magnitude.

This capability boundary restricts vehicle kinematics to an auxiliary role in multisource driver-monitoring systems rather than a standalone warning channel. Comparable signal types may be available from onboard sensing systems without continuous ocular measurement, but their potential value as fallback information when visual sensing is unavailable or degraded was not evaluated in the present study. Table 9 further illustrates the operating-point trade-off. At the default threshold of 0.50, M1 produced approximately 29 false-positive windows per hour but achieved a high-VLI recall of only 0.109. These counts refer to overlapping windows rather than merged operational false-alarm events and therefore do not represent the operational false-alarm rate of a deployed system. The present findings thus motivate evaluating vehicle kinematics as a low-salience trend cue or auxiliary fusion input. Whether sensor fusion or temporal aggregation improves practical driver- or route-level monitoring requires prospective evaluation.

From a deployment perspective, the feature types examined here, including speed, longitudinal and lateral acceleration, angular velocity, and derived jerk, are commonly available or derivable from onboard inertial and chassis sensing. Their window-level computation is lightweight under the 1 Hz, 10 s scheme, suggesting computational feasibility for embedded implementation. However, because the present data were collected using a high-precision inertial navigation system, transfer to production-vehicle CAN bus signals and real-time embedded performance should be validated empirically.

### 4.4. Limitations and Future Work

This study has several limitations. First, the VLI is an operational proxy label constructed from eye-movement statistics and has not been externally validated against subjective scales such as the NASA-TLX or an independent physiological benchmark, so the conclusions apply only to reconstructability within this proxy space and cannot be generalized to cognitive load itself. Second, the dataset contains only 33 drivers on a single fixed 48.4 km round-trip route in one city and is limited to weekday off-peak periods and clear weather. The limited sample size and scenario diversity restrict generalizability, and the two road categories cannot support finer road stratification or interaction analysis. Moreover, because the route contained no formal weaving segments, visual load in such segments remains uncharacterized. Third, the driving-style prior is characterized by only three K-means clusters, and the non-significance of the style increment may partly stem from the sample size; the related conclusions are therefore stated as effect sizes with confidence intervals rather than significance claims and await confirmation in a study with a larger driver sample. Relatedly, the pronounced driver-level heterogeneity in high-VLI proportions (5.2–89.1%) may partly reflect trait-like oculomotor differences rather than differences in visual load. The quantile thresholds were estimated across drivers within each training fold, whereas only the pupil features received per-driver baseline correction. Standardizing all eye-movement features per driver was not adopted because it could also remove meaningful between-driver variation in the VLI proxy. The absence of a driver-standardized sensitivity analysis leaves this source of variation unresolved. Fourth, although the pupil features underwent robust baseline correction, residual illumination effects cannot be fully excluded, though the pupil-removed sensitivity analysis in Table 7 shows that the main conclusions are unaffected. Fifth, the vehicle-kinematic data were acquired with a high-precision inertial navigation system that differs from production CAN-bus signals in noise level and sampling characteristics, so cross-device transfer remains to be verified. Furthermore, the vehicle-kinematic signals were resampled to 1 Hz for multisource alignment, and the raw high-rate inertial records were not retained after the online preprocessing pipeline; therefore, a higher-frequency sensitivity analysis could not be performed. Sub-second jerk and angular-velocity transients may consequently be attenuated, and the reported vehicle-side information content should be interpreted as a conservative estimate. Future studies should retain inertial data at their native sampling rates to assess the frequency sensitivity of jerk and angular-velocity features. They should also determine whether findings obtained from high-precision inertial measurements transfer to CAN-bus signals from production vehicles. Independent subjective or physiological measures of workload are needed to validate and calibrate the VLI in larger driver samples and across diverse routes, cities, and traffic conditions. Future work could further examine finer, independently evaluated representations of driving style and integrate vehicle kinematics with complementary camera-derived cues, steering-wheel-angle signals, and radar-derived range data. These additional inputs may help distinguish context-mediated effects from those mediated by driver state, as discussed in Section 4.2.

## 5. Conclusions

Taking the eye-movement-derived VLI proxy label as its object, this study developed and evaluated a driver-independent framework for cross-source label transfer and measurement-validity diagnosis. The framework comprises four reusable steps: within-fold label construction, driver-grouped cross-validation, a unified-setting comparison between the predictive models and the road-control model, and positive/negative controls with driver-level confidence-interval estimation. Based on the naturalistic driving data of 33 drivers, the main conclusions are as follows.

(1) Vehicle kinematics partially reconstructed the eye-movement-derived VLI label on held-out drivers, with an out-of-fold AUC of 0.589; this reconstruction ability exceeded the binary road-type proxy, with a ΔAUC of 0.042 after road control, whose driver-level 95% confidence interval [0.006, 0.078] excluded zero. The absolute discriminative performance nevertheless remained modest and was insufficient for standalone high-VLI warning.
(2) The driving-style prior provided a directionally consistent, positive but statistically non-significant increment, whose effect was mainly manifested as an overall shift in the driver-level high-VLI base rate. The three-cluster partition underlying this prior is a dataset-specific description of the 33 drivers rather than a general driving-style taxonomy.
(3) The two findings—vehicle reconstructability and the direction of the style increment—showed qualitatively consistent patterns across five model architectures and multiple label-construction schemes.
(4) In future high-VLI state prediction, the vehicle sequence model did not materially outperform the persistence baseline over the evaluated horizons, and both approaches were close to chance level at the 20 s horizon.

In summary, vehicle kinematics retained a measurable cross-source signature of the eye-movement-derived VLI proxy across held-out drivers, beyond the binary road-type proxy. The principal contribution is a confounder-aware framework for evaluating proxy-label transfer rather than a standalone warning model. The results support the prospective evaluation of vehicle kinematics as a complementary sensing input; external criterion validation and multisource deployment studies remain necessary. The framework may also be applicable to other proxy-label sensing settings.

## Figures and Tables

**Table 1:** Sensor specifications and signal-processing parameters.

Data Source	Parameter	Specification
Eye tracker	Sampling rate	60 Hz
	Gaze accuracy	0.5° (nominal); 0.8° (measured) ^1^
	Fixation/saccade segmentation	I-VT algorithm, 30°/s velocity threshold
	Pupil validity criterion	Confidence > 0.9
Inertial navigation system (WTGAHRS3-TTL/232)	Speed accuracy	<0.1 m·s^−1^
	Attitude accuracy	0.2° (roll/pitch); 0.5° (heading)
	Positioning accuracy	<2.5 m
Signal synchronization and resampling	Time reference	GPS time (unified across all sources)
	Resampling	Cubic-spline interpolation to 1 Hz

^1^ Measured after multi-point calibration.

**Table 2:** Traffic operating conditions by road type.

Indicator	Road A: Urban Expressway (80 km/h)	Road B: Freeway (120 km/h)	Overall
Operating speed, mean ± SD (km/h)	50.3 ± 8.6	70.2 ± 11.4	58.7 ± 13.9
Basic-segment mean speed (km/h)	54.1	76.5	—
Operating speed, P15-P85 (km/h)	39.5–61.0	57.6–83.9	43.1–78.2
Mean speed-to-limit ratio	0.63	0.59	—
Severely constrained windows (%)	1.9	0.6	1.3
One-way travel time, mean ± SD (min)	13.9 ± 1.2	10.8 ± 1.0	24.7 ± 1.8
Between-session CV of one-way travel time (%)	8.6	9.3	7.3

Note: Basic segments exclude ramp-influenced zones. Severely constrained windows are defined as windows with a mean speed below 30 km/h. P15–P85 denotes the 15th–85th percentile range; CV, coefficient of variation; “—”, not applicable. Travel-time statistics are computed at the level of individual one-way passes across all retained sessions, including supplementary sessions.

**Table 3:** Definitions and units of the window-level vehicle-kinematic features and the road-context variable.

Feature Name	Symbol	Window-Level Definition	Unit
Speed	*v*	Statistics of speed within the window	m·s^−1^
Longitudinal acceleration	*a_x_*	Statistics of longitudinal acceleration	m·s^−2^
Lateral acceleration	*a_y_*	Statistics of lateral acceleration	m·s^−2^
Jerk	*j*	Statistics of the rate of change of acceleration	m·s^−3^
Angular velocity	*ω*	Statistics of heading and yaw angular velocity	rad·s^−1^
Road-type/segment encoding	*R_i_*	Road A/B encoding, by window-midpoint position	categorical

Note: All features are computed within a 10 s sliding window; for each vehicle quantity, five window-level statistics (mean, standard deviation, maximum, minimum, and median) are computed.

**Table 4:** Weights of the WESF features in VLI label construction.

Feature	Category	Entropy Weight	CRITIC Weight	Combined Weight
Number of fixations	Fixation	0.0734	0.1131	0.0922
SD of fixation duration	Fixation	0.0980	0.1173	0.1071
Total number of saccades	Saccade	0.1254	0.1158	0.1209
Total saccade time	Saccade	0.1597	0.1138	0.1380
SD of saccade time	Saccade	0.2239	0.0946	0.1627
Mean saccade amplitude	Saccade	0.1047	0.0918	0.0986
Mean pupil diameter	Pupil	0.1115	0.2194	0.1636
SD of pupil diameter	Pupil	0.0566	0.0642	0.0602
CV of pupil diameter	Pupil	0.0448	0.0701	0.0568

Note: All nine WESF features have a positive direction and all enter the primary VLI; the pupil features are counted only in valid windows with pupil confidence >0.9. Weights shown are five-fold means; fold-wise values are given in Table A8.

**Table 5:** Input settings of the predictive model and their road-confounding-control roles.

Setting	Input	Role	Interpretive Purpose
M0 Road-only	Road type	Confounding baseline	Assess the discriminative ability of the road proxy itself
M1 Vehicle-only	Vehicle-kinematic window sequence	Primary model 1	Test whether the vehicle modality can predict the VLI label
M2 Vehicle+Style	Vehicle sequence + within-fold style prior	Primary model 2	Test the driving-style increment
M3 Road+Vehicle	Road type + vehicle kinematics	Road control	Test whether the vehicle exceeds the road proxy
M4 Road+Vehicle+Style	Road + vehicle + style prior	Sensitivity analysis	Test the style increment after road control

**Table 6:** VLI label distribution and driver-level high-VLI proportions.

Distribution Item	Full Sample	Road A	Road B	Driver-Level Range
Number of windows	24,437	13,789	10,648	573–974
Low-VLI proportion	33.2%	28.1%	39.9%	0.0–81.3%
Medium-VLI proportion	33.4%	33.9%	32.8%	9.2–50.6%
High-VLI proportion	33.3%	38.0%	27.3%	5.2–89.1%
VLI mean	0.214	0.223	0.202	0.120–0.351
VLI median	0.207	0.217	0.194	0.086–0.363
Road A proportion	56.4%	—	—	49.7–63.7%
Road B proportion	43.6%	—	—	36.3–50.3%

Note: Dashes (—) indicate entries not applicable to the corresponding column; the driver-level range gives the minimum and maximum across the 33 drivers.

**Table 7:** VLI label agreement rate and predictive stability under alternative weighting schemes and the pupil-removed variant.

VLI Construction Method	Label Agreement	Vehicle-Only AUC	Vehicle+Style AUC	ΔAUC (Style)
Combined weight (primary VLI)	100.0%	0.589	0.633	0.044
Entropy weighting	90.9%	0.570	0.595	0.025
CRITIC	90.6%	0.617	0.669	0.052
Equal weighting	93.3%	0.580	0.614	0.033
PCA-1	87.6%	0.596	0.610	0.014
Pupil-removed combined VLI	73.3%	0.562	0.564	0.003

**Table 8:** Out-of-fold discriminative performance and style increment for each input setting.

Input Setting	Acc.	Macro-F1	High-VLI Recall	AUC	AP	ΔAUC vs. Veh.	95% CI
Vehicle-only	0.673	0.489	0.109	0.589	0.430	—	—
Vehicle+Style	0.690	0.520	0.143	0.633	0.490	+0.044	[−0.005, 0.113]
Style-only	0.688	0.472	0.072	0.532	0.451	−0.058	[−0.188, 0.074]
Road-only	0.667	0.400	0.000	0.562	0.400	−0.027	[−0.070, 0.032]
Road+Vehicle	0.682	0.496	0.118	0.604	0.442	+0.015	[−0.035, 0.065]
Road+Vehicle+Style	0.698	0.531	0.155	0.642	0.504	+0.053	[0.013, 0.093]

**Table 9:** Out-of-fold operating points for high-VLI detection with training-fold-selected thresholds.

Model	Threshold Policy (Training Folds Only)	Precision	Recall	FPR	FPW/h
M1 Vehicle-only	Default 0.50	0.48	0.109	0.060	29
M1 Vehicle-only	Max-F1	0.41	0.42	0.302	145
M1 Vehicle-only	Recall ≥ 0.50	0.39	0.51	0.398	191
M1 Vehicle-only	Recall ≥ 0.70	0.37	0.71	0.604	290
M2 Vehicle+Style	Default 0.50	0.53	0.143	0.063	30
M2 Vehicle+Style	Max-F1	0.44	0.47	0.299	143
M2 Vehicle+Style	Recall ≥ 0.50	0.43	0.53	0.351	168
M2 Vehicle+Style	Recall ≥ 0.70	0.40	0.72	0.539	259

Note: Thresholds were selected on training folds only. Max-F1 maximizes F1; FPW/h denotes false-positive windows per hour calculated using Equation (9).

**Table 10:** Driver-level vehicle-kinematic style-cluster centers and sample distribution.

Style Cluster	No. of Drivers	Mean Speed (m·s^−1^)	Speed Fluctuation (m·s^−1^)	Mean Long. Accel. (m·s^−2^)	Long. Accel. Fluct. (m·s^−2^)	Mean Abs. Jerk (m·s^−3^)
Style 1	13	14.837	0.125	0.352	0.296	0.138
Style 2	14	16.700	0.160	−0.025	0.323	0.195
Style 3	6	18.627	0.104	0.449	0.338	0.114

**Table 11:** AUC and style increment across the five model architectures.

Model	Vehicle AUC	Vehicle+Style AUC	Style Increment ΔAUC	High-VLI Recall (V → V+S)
Logistic	0.589	0.633	+0.044	0.109 → 0.143
RF	0.624	0.632	+0.008	0.160 → 0.186
XGBoost	0.570	0.587	+0.017	0.085 → 0.123
GRU	0.600	0.642	+0.042	0.161 → 0.226
Transformer-GRU	0.599	0.639	+0.040	0.121 → 0.235

Note: The logistic row is consistent with Table 8 and serves as an anchor for cross-checking. The increments for each architecture are compared descriptively only; interval inference is given in Section 3.5.

**Table 12:** Mean absolute SHAP importance and direction of influence of the main vehicle response features.

Rank	Feature	Sensing Source	Mean |SHAP| (Proportion)	Direction
1	Angular-velocity statistics	Vehicle kinematics	0.293	Positive
2	Speed statistics	Vehicle kinematics	0.232	Negative
3	Driving-style cluster	Vehicle-derived style	0.223	Cluster-dependent (n.s.)
4	Longitudinal-acceleration statistics	Vehicle kinematics	0.140	Positive
5	Lateral-acceleration statistics	Vehicle kinematics	0.087	Direction unclear
6	Jerk statistics	Vehicle kinematics	0.025	Positive

Note: Proportions are normalized mean absolute SHAP values and sum to 1. Direction is based on fold-wise coefficient signs; inconsistent signs or near-zero coefficients are labeled unclear.

**Table 13:** Increment-test results under road-confounding control.

Comparison	Test Content	ΔAUC	Driver-Level 95% CI
M2 − M1 (road not controlled)	Style increment	+0.044	[−0.005, 0.113]
M3 − M0	Vehicle increment over road	+0.042	[0.006, 0.078]
M4 − M3	Style increment after road control	+0.038	[−0.012, 0.088]

**Table 14:** VLI distribution and out-of-fold model performance by window category.

Maneuver Category	Windows (*n*, %)	VLI Mean	High-VLI Proportion (%)	M1 AUC	M2 AUC	Mean Predicted High-VLI Probability (M2)
Merge/diverge	2650 (10.8%)	0.247	45.5	0.603	0.641	0.41
Lane change/overtaking	1140 (4.7%)	0.238	42.0	0.596	0.633	0.38
Regular driving	20,647 (84.5%)	0.207	31.3	0.571	0.612	0.31

## Data Availability

The original contributions presented in this study are included in this article. Further inquiries can be directed to the corresponding author.

## Appendix A

### Appendix A.1. Model Interpretation and Illustrative Results

**Table A1:** Full metrics of the model-invariance check.

Model	Input	Accuracy	Macro-F1	High-VLI Recall	AUC	AP
Logistic	Vehicle	0.673	0.489	0.109	0.589	0.430
RF		0.684	0.526	0.160	0.624	0.478
XGBoost		0.684	0.479	0.085	0.570	0.455
GRU		0.673	0.519	0.161	0.600	0.442
Transformer-GRU		0.667	0.493	0.121	0.599	0.435
Logistic	Vehicle+Style	0.690	0.520	0.143	0.633	0.490
RF		0.696	0.549	0.186	0.632	0.510
XGBoost		0.697	0.512	0.123	0.587	0.485
GRU		0.696	0.567	0.226	0.642	0.500
Transformer-GRU		0.699	0.573	0.235	0.639	0.507

Note: This table is the full-metric version of Table 11 in the main text; the values are identical to the original evaluation results. The logistic rows exactly match the main results in Table 8 and serve as anchor checks.

### Appendix A.2. Context, Direction, and Window-Scheme Sensitivity Analyses

**Table A2:** Segment-level road-context control analysis.

Model	Road-Context Encoding	Vehicle Features	AUC	ΔAUC [95% CI]
M0	Binary road type	No	0.562	—
M3	Binary road type	Yes	0.604	+0.042 [0.006, 0.078]
M0′	Segment ID (14 segments, one-hot)	No	0.581	—
M3′	Segment ID (14 segments, one-hot)	Yes	0.615	+0.034 [0.003, 0.065]

Note: Segments are defined in Section 3.5. ΔAUC (M3 − M0; M3′ − M0′) is reported with driver-level bootstrap 95% CIs; reference rows repeat Table 8 and Table 13. —, not applicable.

**Table A3:** (**a**) Window spatial categories and descriptive statistics. (**b**) Sensitivity of the main results to the spatial classification.

(**a**)					
**Window Category**	**Windows (*n*, %)**	**VLI Mean**	**High-VLI** **Proportion (%)**	**Mean Speed** **(km/h** **)**	**Mean |Angular Velocity|** **(rad/s)**
Basic segment, Road A	11,650 (47.7%)	0.219	36.5	54.1	0.021
Basic segment, Road B	10,050 (41.1%)	0.198	26.6	76.5	0.012
Ramp-influenced zone	2300 (9.4%)	0.246	45.2	45.8	0.038
Connecting ramp	350 (1.4%)	0.252	47.4	38.2	0.055
Boundary-crossing	87 (0.4%)	0.231	39.1	49.6	0.032
(**b**)					
**Setting**	**M1 AUC**	**M2 AUC**	**Road-Control AUC** **(M0 Variant)**	**Vehicle Increment** **ΔAUC [95% CI]**	**Style Increment** **ΔAUC [95% CI]**
Primary analysis	0.589	0.633	0.562	+0.042 [0.006, 0.078]	+0.044 [−0.005, 0.113]
Excluding boundary-crossing and connecting-ramp windows	0.587	0.631	0.561	+0.041 [0.005, 0.077]	+0.043 [−0.006, 0.111]
Three-category road coding R ∈ {A, B, Ramp}	0.589	0.633	0.571	+0.037 [0.004, 0.070]	+0.044 [−0.005, 0.113]
Basic-segment-only subsample	0.578	0.619	0.549	+0.036 [0.002, 0.070]	+0.040 [−0.008, 0.088]
Ramp-influenced-zone-only subsample	0.601	0.634	—	—	—

Note: Window categories are defined in Section 2.1.5. In the three-category setting the road input of M0/M3/M4 is replaced by the {A, B, Ramp} encoding; subsample rows re-estimate all within-fold parameters on the corresponding training-fold subsets.

**Table A4:** Directional sensitivity analysis. (**a**) Descriptive statistics by direction and road type. (**b**) Direction-controlled and direction-stratified evaluation.

(**a**)				
**Direction × Road**	**Windows (*n*)**	**Mean Speed (km/h)**	**VLI Mean**	**High-VLI Proportion (%)**
Outbound, Road A	6950	49.8	0.225	38.9
Outbound, Road B	5310	71.1	0.204	27.9
Return, Road A	6839	50.9	0.221	37.1
Return, Road B	5338	69.4	0.200	26.7
(**b**)				
**Setting**		**AUC/ΔAUC**	**Driver-Level 95% CI**	
M0″ Road × Direction only		0.566	—	
M3″ Road × Direction + vehicle		0.606	—	
Vehicle increment M3″ − M0″		+0.040	[0.005, 0.075]	
M1 AUC, outbound/return strata		0.585/0.592	—	
M2 AUC, outbound/return strata		0.628/0.636	—	

**Table A5:** High-VLI prediction with strictly non-overlapping windows.

Horizon	Persistence Baseline	M0 Road-Only	M1 Vehicle-Only	M2 Vehicle+Style	Vehicle Sequence Model
10 s	0.703	0.548	0.566	0.588	0.574
20 s	0.531	0.541	0.552	0.569	0.560
30 s	0.505	0.536	0.541	0.552	0.549

**Table A6:** Sensitivity of the driving-style prior to road- and traffic-related components of the style features.

Style Construction	K	Mean Silhouette	M1 AUC	M2 AUC	Style Increment M2 − M1 ΔAUC [95% CI]	M4 − M3 ΔAUC [95% CI]
Primary	3	0.450	0.589	0.633	+0.044 [−0.005, 0.113]	+0.038 [−0.012, 0.088]
Mean speed removed from clustering	3	0.402	0.589	0.621	+0.032 [−0.014, 0.089]	+0.028 [−0.019, 0.075]
Segment-referenced deviation features	3	0.418	0.589	0.626	+0.037 [−0.010, 0.096]	+0.031 [−0.016, 0.081]

Note: The segment-referenced construction follows Equation (7); M1 does not involve the style prior and is repeated as reference.

### Appendix A.3. Cross-Validation and Label-Construction Reproducibility

Table A7 lists the composition of the five driver-grouped cross-validation folds. All sessions of a given driver, including supplementary sessions (Section 2.1.4), were assigned to the same fold, so no driver contributed data to both a training and a held-out partition.

**Table A7:** Fold composition under driver-grouped five-fold cross-validation.

Fold	Drivers (*n*)	Driver IDs	Drivers with Two Sessions (*n*)
1	6	D05, D10, D13, D16, D22, D25	1
2	6	D09, D15, D19, D26, D30, D33	1
3	7	D02, D04, D06, D07, D18, D27, D29	2
4	7	D08, D12, D14, D21, D23, D28, D31	2
5	7	D01, D03, D11, D17, D20, D24, D32	2
Total	33	—	**8**

Note: Driver IDs are anonymized codes (D01–D33).

Driver-level bootstrap confidence intervals (B = 200) for the increment contrasts were computed with the percentile method: drivers were resampled with replacement over the fixed out-of-fold predictions, the increment statistic was recomputed for each resample, and the 2.5th and 97.5th percentiles of the bootstrap distribution were taken as the interval bounds. All stochastic components used fixed random seeds recorded in the analysis code: master fixture seed 4481733; K-means initialization seeds 4481834–4481838 (n_init = 100); percentile bootstrap seed 4482733; random-representation control seeds 4483995–4484094; tree-model fold seeds 4485111–4485325; GRU fold seeds 4487101–4487205; and Transformer-GRU fold seeds 4488101–4488205.

Table A8 reports the combined entropy–CRITIC weight of each of the nine WESF features as estimated within each of the five training folds; the weights reported in Table 4 are the corresponding five-fold means, and fold-to-fold variation is small.

**Table A8:** Combined WESF weights by training fold.

Feature	Fold 1	Fold 2	Fold 3	Fold 4	Fold 5	Mean ± SD
Number of fixations	0.0938	0.0899	0.0915	0.0938	0.0920	0.0922 ± 0.0017
SD of fixation duration	0.1070	0.1078	0.1075	0.1068	0.1063	0.1071 ± 0.0006
Total number of saccades	0.1229	0.1185	0.1217	0.1198	0.1216	0.1209 ± 0.0018
Total saccade time	0.1383	0.1384	0.1372	0.1378	0.1381	0.1380 ± 0.0005
SD of saccade time	0.1621	0.1639	0.1618	0.1626	0.1631	0.1627 ± 0.0008
Mean saccade amplitude	0.0967	0.0999	0.0989	0.0988	0.0985	0.0986 ± 0.0012
Mean pupil diameter	0.1615	0.1640	0.1649	0.1636	0.1639	0.1636 ± 0.0013
SD of pupil diameter	0.0588	0.0614	0.0592	0.0611	0.0605	0.0602 ± 0.0011
CV of pupil diameter	0.0589	0.0562	0.0573	0.0557	0.0559	0.0568 ± 0.0013

Held-out feature values were not clipped. After training-fold min–max normalization, values outside [0, 1] occurred in 0.049% of held-out windows (12/24,437) and were used as is; because the VLI enters the analysis through its within-fold tercile threshold, these values affect label assignment only at the extremes.

The random-representation negative control was repeated 100 times with independent driver-level random draws (base seed 4,483,995; repetition r used seed 4483995 + r − 1), each following the identical frozen driver-grouped five-fold protocol. For each repetition, the AUCs of the five held-out folds were averaged; across repetitions, the held-out AUC was 0.4982 ± 0.0633 (range 0.3334–0.6561). The value of 0.498 reported in Section 3.2 corresponds to the mean across all 100 repetitions.

Table A9 summarizes the implementation of the five predictive models and the auxiliary K-means style prior. RF and XGBoost hyperparameters were selected by nested driver-grouped cross-validation within the training folds, whereas the GRU and Transformer-GRU configurations were fixed before evaluation.

### Appendix A.4. Model Implementation and Hyperparameter Selection

**Table A9:** Model configurations, training settings, and hyperparameter-selection procedure.

Model	Parameter	Value
Logistic regression (primary model, M0–M4)	Penalty	L2 (ridge)
	Regularization strength	C = 1000 (λ = 0.001; intercept unpenalized)
	Solver	Newton–Raphson/IRLS
	Class weight	None
	Max iterations	12 (convergence tolerance = 1 × 10^−7^)
Random forest	Number of trees (n_estimators)	300
	Max depth	8
	Min samples per leaf	10
	Max features	sqrt(p)
	Class weight	balanced
XGBoost	n_estimators	100
	max_depth	1
	learning_rate	0.01
	subsample	0.8
	colsample_bytree	0.8
	reg_lambda/reg_alpha	1.0/0.0
	scale_pos_weight	n_negative/n_positive within each training fold (≈2.0)
GRU	Sequence length	10 windows
	Layers	1 GRU layer
	Hidden size	32
	Dropout	0.20 (post-GRU)
	Optimizer	Adam
	Learning rate	0.001
	Batch size	512
	Max epochs	6
	Early stopping	Validation loss; patience = 2 validation checks; validation every 20 mini-batches
	Loss function	Class-weighted cross-entropy
Transformer-GRU	Sequence length	10 windows
	Positional embedding	Learned; dimension = 32
	Self-attention blocks	1
	Attention heads	4
	Model dimension	32
	GRU hidden size	32
	Dropout	Attention 0.10; post-attention 0.20; post-GRU 0.20
	Optimizer	Adam
	Learning rate	0.001
	Batch size	512
	Max epochs	6
	Early stopping	Validation loss; patience = 2 validation checks; validation every 20 mini-batches
	Loss function	Class-weighted cross-entropy
K-means style prior	K (pre-specified)	3 (fixed before the modeling analysis)
	Initialization	k-means++
	n_init	100
	Max iterations	1000
Hyperparameter selection	Search space	RF: 300 trees; (max_depth, min_samples_leaf) = (4, 40), (6, 20), (8, 10), (10, 5), or (None, 10); max_features = sqrt. XGBoost: (n_estimators, max_depth, learning_rate) = (100, 1, 0.01), (200, 1, 0.03), (300, 1, 0.03), (200, 2, 0.01), (300, 2, 0.03), or (300, 3, 0.03). GRU and Transformer-GRU used the fixed configurations reported above.
	Selection metric	Mean inner-fold ROC-AUC, maximized
	Inner validation scheme	Nested driver-grouped cross-validation: for each outer fold, the remaining four driver folds were rotated as inner validation folds; the selected configuration was refitted on all four outer-training folds and evaluated once on the held-out outer fold

Note: The scale_pos_weight parameter was recalculated within each XGBoost training fold; GRU and Transformer-GRU required no inner hyperparameter search.
